# Persistent Properties of a Subpopulation of Cancer Cells Overexpressing the Hedgehog Receptor Patched

**DOI:** 10.3390/pharmaceutics14050988

**Published:** 2022-05-05

**Authors:** Álvaro Javier Feliz Morel, Anida Hasanovic, Aurélie Morin, Chloé Prunier, Virginie Magnone, Kevin Lebrigand, Amaury Aouad, Sarah Cogoluegnes, Judith Favier, Claude Pasquier, Isabelle Mus-Veteau

**Affiliations:** 1 Université Côte d’Azur, CNRS, Institut de Pharmacologie Moléculaire et Cellulaire (IPMC), 06560 Valbonne, France; javitto28@gmail.com (Á.J.F.M.); anida160@hotmail.com (A.H.); magnone@ipmc.cnrs.fr (V.M.); lebrigand@ipmc.cnrs.fr (K.L.); amaury.aouad@univ-cotedazur.fr (A.A.); cogoluegnes@ipmc.cnrs.fr (S.C.); 2 Université de Paris, PARCC, INSERM, Equipe Labellisée par la Ligue Contre le Cancer, CEDEX 15, 75737 Paris, France; aurelie.morin@gmail.com (A.M.); judith.favier@inserm.fr (J.F.); 3 INOVOTION, Biopolis-5 Av. du Grand Sablon, 38700 La Tronche, France; chloe.prunier@inovotion.com; 4 Université Côte d’Azur, CNRS-UMR7271, Laboratoire d’Informatique, Signaux et Systèmes de Sophia Antipolis (I3S), 06560 Valbonne, France; claude.pasquier@univ-cotedazur.fr

**Keywords:** Patched, chemotherapy resistance, metastases, cancer stem cells, persistent cells, adrenocortical carcinoma

## Abstract

Despite the development of new therapeutic strategies, cancer remains one of the leading causes of mortality worldwide. One of the current major challenges is the resistance of cancers to chemotherapy treatments inducing metastases and relapse of the tumor. The Hedgehog receptor Patched (Ptch1) is overexpressed in many types of cancers. We showed that Ptch1 contributes to the efflux of doxorubicin and plays an important role in the resistance to chemotherapy in adrenocortical carcinoma (ACC), a rare cancer which presents strong resistance to the standard of care chemotherapy treatment. In the present study, we isolated and characterized a subpopulation of the ACC cell line H295R in which Ptch1 is overexpressed and more present at the cell surface. This cell subpopulation is more resistant to doxorubicin, grows as spheroids, and has a greater capability of clonogenicity, migration, and invasion than the parental cells. Xenograft experiments performed in mice and in ovo showed that this cell subpopulation is more tumorigenic and metastatic than the parental cells. These results suggest that this cell subpopulation has cancer stem-like or persistent cell properties which were strengthened by RNA-seq. If present in tumors from ACC patients, these cells could be responsible for therapy resistance, relapse, and metastases.

## 1. Introduction

Cancer development is a complex process combining mutational accumulation and dynamic changes. Tumors usually involve heterogeneous cell populations (stem cells, progenitors or differentiated tumor cells) with functionally divergent phenotypes (mitotic or not, migratory or static, pro-angiogenic or not) [1,2]. Such heterogeneity underlies the lethal outcome of cancer, therapeutic failure, and drug resistance [3,4]. Cancer stem cells (CSCs) are of particular interest in this context. They are a subpopulation of tumor cells with unlimited self-renewal potential, capable of giving rise to all tumor cell types within a tumor, and resistant to many conventional anticancer treatments that affect more differentiated tumor cells. In 2018, 18.1 million cancers were diagnosed worldwide and 9.6 million people died. These deaths were often the result of recurrences or metastases due to the resistance of cancer cells to the chemotherapy treatments used and the lack of response to immunotherapy.

The resistance of cancer cells to chemotherapy is therefore one of the major challenges in the clinical management of cancer. This phenomenon called multidrug resistance (MDR) induces less sensitivity of cancer cells to classical and targeted chemotherapies, and has been intensively studied. Many mechanisms such as increased DNA damage repair, modification or alteration of drug or target proteins, drug efflux, reduced apoptosis, hypoxia or transformation of epithelial cells to mesenchymal cells can induce MDR [5,6].

Drug efflux induced by the overexpression of the ATP-binding cassette (ABC) transporters has been considered the most prominent underlying mechanism for MDR [7]. Since the discovery of the P-glycoprotein (Pgp or MDR1) over 35 years ago, some studies have linked ABC transporter expression to poor outcome in several cancer types, leading to the development of Pgp inhibitors tested in clinical trials to overcome MDR [8]. At least three generations of Pgp inhibitors have been tested. All these compounds failed in clinical trials due to lack of potency, off-target effects or toxicity issues. Members of the ABC superfamily transport toxins, sugars, amino acids, nucleotides and metabolites out of cells, and protects cells against toxic molecules, including drugs with very different chemical structures [9]. Thus, ABC transporters are particularly important for the functioning of healthy cells, and that is why, to date, no inhibitors of ABC transporters have obtained approval from FDA [10,11]. Therefore, a treatment able to overcome chemotherapy resistance and then eliminate the resistant cancer cells responsible for the relapse and metastases (also called persister cells) is still an unmet, urgent medical need.

The Hedgehog (Hh) signaling pathway has a crucial role during early embryonic development, controlling cell differentiation and proliferation. In adults, this pathway regulates stem cell homeostasis and tissue regeneration. The Hh signaling pathway activation has been correlated with an increase in cancer development, progression and metastasis. Indeed, many aggressive cancers present an aberrant activation of the Hh signaling pathway [12], particularly in cells that present a chemotherapy resistance, i.e., cancer stem cells (tumor-initiating cells/persistent cells) [13]. The expression of the Hh receptor, Patched (Ptch1), is expressed upon activation of the Hh pathway. This receptor is overexpressed in many primary cancers (i.e., brain, breast, colon, melanoma, prostate and ovary) (see [14] for a review), and some studies proposed Ptch1 as an early marker of thyroid and gastric cancers [15,16].

We have discovered that Ptch1 is a multidrug transporter that contributes to the efflux of chemotherapeutic agents and plays an important role in the resistance to chemotherapy in adrenocortical carcinoma and melanoma cells [17,18,19]. Adrenocortical carcinoma (ACC) is a rare cancer which presents strong resistance to the most efficient available treatment composed of a mixture of chemotherapeutic agents (etoposide, doxorubicin and cisplatin) combined with the adrenolytic substance mitotane (EDP-M) [20,21]. Our analyses showed that Ptch1 was present in primary tumor samples from the 70 ACC patients of the cohort studied [18]. Decreasing endogenous Ptch1 expression using siRNA strongly inhibited the efflux of doxorubicin (dxr) from ACC cells indicating that Ptch1 is involved in dxr efflux in ACC cells. Accordingly, we showed that ACC cells rendered resistant to dxr express more Ptch1 than parental cells.

In adults, Ptch1 is poorly expressed in healthy cells and functions as a drug efflux pump only in cancer cells. Indeed, Ptch1 activity uses the proton gradient as an energy source and transports chemotherapeutic agents out of the cells against a proton entry. This requires the extracellular medium to be more acidic than the intracellular medium, which is a characteristic of cancer cells known as the Warburg effect [22]. Indeed, the high glucose consumption of cancer cells causes the extracellular medium to become more acidic and allows Ptch1 to drive the chemotherapeutic agents out of the cells. This makes Ptch1 an innovative and highly promising therapeutic target to improve the effectiveness of anticancer chemotherapeutic treatments without toxicity for healthy cells.

We have developed screening tests to identify molecules that inhibit the resistance to dxr conferred by human Ptch1 to yeast, and the efflux of dxr by Ptch1 [23]. This led to the discovery of three inhibitors of Ptch1 drug efflux. Methiothepin, a drug-like compound antagonist of the serotonin receptor, increases the efficacy of dxr against ACC cells in vitro and in vivo [18], and of dxr and vemurafenib against melanoma cells [24]; Astemizole, a non-sedating antihistaminergic drug increases the efficacy of dxr and cisplatin against ACC cells in vitro [25]; and Panicein A hydroquinone (PAH) produced by marine sponges increases the efficacy of dxr and cisplatin against melanoma cells in vitro [26], and of vemurafenib against BRAF^V600E^ melanoma cells in vitro and in vivo [19].

In the present study, we isolated and characterized a subpopulation of the ACC cell line H295R in which Ptch1 is overexpressed and more present at the cell plasma membrane. The in vitro, in vivo and RNA-seq studies performed reveal that these cells are more resistant to chemotherapy than the parental cell line and have cancer stem-like or persistent cell properties.

## 2. Materials and Methods

### 2.1. Chemical and Biological Material

Doxorubicin hydrochloride (dxr) was purchased from Sigma-Aldrich, Burlington, MA, USA, Methiothepin maleate (P375) was purchased from Santa Cruz, CA, USA: CAS number: 20229-30-5; MW: 472.62; molecular formula: C20H24N2S2.C4H4O4.

The human adrenocortical carcinoma cell line H295R and H295R-PM-Ptc+ were cultured in DMEM/F12 medium supplemented with 2% of NuSerum (BD), 1% ITS (1.0 mg/mL recombinant human insulin, 0.55 mg/mL human transferrin (substantially iron-free), and 0.5 g/mL sodium selenite, 100×, BD), 1% GlutaMAX (Gibco), Penicillin/Streptomycin 1% 100x (Gibco). H295RdxrR was obtained by adding increasing concentrations of doxorubicin up to 0.2μM in the culture medium over 6 months. Cells were cultured in Falcon flasks or plates (Corning, Inc., Corning, NY, USA) at 37 °C and 5% CO_2_/95% air water-saturated atmosphere.

### 2.2. Flow Cytometry

Cells were collected using Accutase (StemCell), centrifuged and incubated with monoclonal rat anti-Ptch1 antibody (MAB41051 R&D Systems; 10 µg/mL) and then with anti-rat antibody coupled to Alexa 594 in ice in FACS buffer (PBS buffer with FBS 5% and EDTA 2 µM). FACS analyses were performed using DB LSRFortessa (BioSciences). Sorting of H295R-PM-Ptc+ was performed using BD FACSAria III.

### 2.3. Immunofluorescence

Cells were seeded on coverslips in 24-well plates and allowed to grow to 80% confluence. Cells were fixed with 1% paraformaldehyde (PFA), incubated for 2 h on ice with rat anti-Ptch1 antibody (MAB41051 R&D Systems; 10 µg/mL) and then with anti-rat antibody coupled to Alexa 594 in PBS buffer with 0.1% BSA. Images were acquired with a Zeiss Axioplan 2 fluorescence microscope coupled to a digital charge-coupled device camera using a 40×/1.3 Plan NeoFluar objective and filter for Alexa 594.

### 2.4. Western-Blot

Western-blots were performed using standard techniques. Cells were lysed using RIPA (radioimmunoprecipitation assay) buffer. Total protein amount from cell lysates was determined using DC Protein Assay (500–0116; BioRad, Hercules, CA, USA). 80 µg of total extract was loaded per sample and separated on an 8% SDS-PAGE gel, then transferred to nitrocellulose membrane (Amersham). Unspecific binding was reduced by incubating the membranes in blocking buffer (20 mM Tris-HCl pH 7.5, 45 mM NaCl, Tween 20 0.1% and non-fat milk 5%) for 1 h at room temperature. Nitrocellulose membranes were then incubated overnight at 4 °C with rat anti-Ptch1 antibody (MAB41051 R&D Systems; 1 µg/mL), rabbit anti-ADCY2 (Abnova) (1/1000), rabbit anti-Gli2 (Abcam) (1/1000), rabbit anti-ABCG2 (Genetex) (1/500), rabbit anti-GRK5 (Genetex) (1/1000), rabbit anti-SLUG (SNAI2) (Genetex) (1/2500), rabbit anti-SOX5 (Genetex) (1/1000), rabbit anti-Nanog (Cell Signaling Technology) (1/1000), and mouse anti-tubulin (Sigma) (1/1000) or rabbit anti-GAPDH (Abcam ab37168) (1/10,000). After 3 washes, membranes were incubated with the corresponding horseradish peroxidase (HRP)-coupled secondary antibody for 45 min (goat anti-rat IgG HRP conjugate (Life Technologies; 1/2000), goat anti-rabbit IgG HRP conjugate (Invitrogen, 1/5000) or goat anti-mouse IgG HRP conjugate (Dako, 1/5000)). HRP signal was revealed using an ECL Prime Western blotting detection reagent (RPN2232; Amersham) and detected using a Fusion FX Imager (Vilber Lourmat).

### 2.5. Cytotoxicity Assay

Cytotoxicity assays were performed as described in [18]. Cells were grown in 96-well plates until 70–80% of confluence before replacing medium with 100 µL of complete medium containing methiothepin or DMSO as control (methiothepin solvent). Following an incubation of 2 h at 37 °C and 5% CO_2_, 100 µL of medium containing increasing concentrations of doxorubicin (dxr) were added to the cells. After 48 h incubation at 37 °C and 5% CO_2_, the cytotoxic effect of dxr was addressed using neutral red assay (NR). NR was diluted in complete medium (50 µg/mL), and cells were incubated for 3 h in 100 µL of NR solution at 37 °C 5% CO_2_. NR incorporated into living cells by lysosomes was measured using a Multiskan Go Microplate Spectrophotometer (Thermo Scientific, Waltham, MA, USA). IC50 was calculated using GraphPad Prism 6 software.

### 2.6. Doxorubicin Accumulation in Cells

Cells were seeded on coverslips placed in 24-well plates. Once cells reached 80% of confluence, the cells’ medium was replaced with a physiological buffer (140 mM NaCl, 5 mM KCl, 1 mM CaCl_2_, 1 mM MgSO_4_, 5 mM glucose, 20 mM HEPES, pH 7.4) containing 2 µM of doxorubicin (dxr). After different incubation time points (15, 30, 60, 180 and 240 min), coverslips were fixed with PFA 4% in PBS, washed with PBS and mounted using a SlowFade Gold antifade reagent containing DAPI (Invitrogen). Images of the coverslips were taken using a Zeiss Axioplan 2 fluorescence microscope coupled to a charge-coupled device camera using a 40×/1.3 Plan NeoFluar objective and filters for Alexa 594. Dxr fluorescence was measured using ImageJ software 2015. Around 100 cells (selection of cells was performed randomly) from three different coverslips were measured for each time point and condition of the experiment.

### 2.7. Clonogenic Assay

Five thousand cells were seeded in 24 well plates. After 14 days, cells were fixed using PFA 4% and then colored using crystal violet 0.4%. Pictures of the colored colonies were taken, and crystal violet was solubilized in PBS buffer containing SDS 2%. The absorbance at 620 nm of the solubilized crystal violet was measured using Multiskan Go Microplate Spectrophotometer (Thermo Fisher Scientific, Waltham, MA, USA).

### 2.8. Transwell Invasion Assay

Invasion was measured using a CytoSelect 24-well cell invasion assay according to the manufacturer’s instructions (Cell Biolabs). Briefly, 3 × 10^5^ cells were seeded in the upper chamber of the transwell in complete culture medium, and complete culture medium was added in the lower chamber. The plate was incubated at 37 °C in 5% CO_2_ for 6 days to induce invasion towards the lower chamber. Noninvasive cells were removed from the upper side of the chamber, and invasive cells were stained and quantified by optical density at 560 nm using a Multiskan Go Microplate Spectrophotometer (Thermo Scientific, Waltham, MA, USA). Obtained values were normalized to the number of cells (from triplicate wells).

### 2.9. Wound-Healing Assay

Cells were seeded in 24 well plates in complete culture medium. When cells formed a confluent carpet, a wound was performed with a p200 tip. Two pictures were taken at two different points of the wound for each well. The images were taken every 15 min during 24 h using a Cytation 5 Cell Imaging Multi-Mode Reader (Biotek, Agilent, Santa Clara, CA, USA). Images were analyzed using a macro for ImageJ developed by MICA (Microscopie Imagerie Côte d’Azur) and the reclosing slope was calculated. Migration was measured as the wound area decrease for 24 h (pixel^2^/time). Since cells could not completely reclose the wound within 24 h, cells were kept in culture, and images were taken 12 and 20 days after the wound, using Leica DM IRB (5×).

### 2.10. In ovo Experiment

In ovo experiments were performed by INOVOTION SAS (Biopolis, 5 avenue du grand sablon, La Tronche, 38700, France–STU20210623).

Fertilized White Leghorn eggs were incubated at 37 °C with 50% relative humidity for 9 days. On day 9, the chorioallantoic membrane (CAM) was dropped down by drilling a small hole through the eggshell into the air sac, and a 1 cm^2^ window was cut in the eggshell above the CAM. Then, 3 × 10^6^ H295R or H295R-PM-Ptc+ cells were inoculated onto the CAM of each egg and the eggs were randomized into groups. On day E18, the upper portion of the CAM (with tumor) was removed, washed with PBS buffer and then fixed in PFA for 48 h. After that, tumors were carefully cut away from normal CAM tissue and weighed. Chicken embryos livers were collected (10 livers per group). Genomic DNA was extracted using a commercial kit and analyzed by real time qPCR with specific primer for Human Alu sequences [27]. Calculation of Cq for each sample, mean Cq and relative amounts of metastases for each group were directly managed by the Bio-Rad CFX Maestro software 2.0.

### 2.11. Experiments in Mice

Seven-week-old immunodeficient NMRI Nude mice were injected in renal subcapsular with 3 × 10^6^ H295R or H295R-PM-Ptc+ cells in 40 µL of PBS/matrigel 1:1 v:v. Ten mice received H295R parental cells and 10 mice received H295R-PM-Ptc+ cells. Mice were sacrificed 45 days after cell injection, and the tumors were collected, weighed, fixed and included in paraffin. Project CEEA 17-108 accepted by the ethic committee under number MESR 15476.

### 2.12. Immunofluorescence on Mouse Tumor Slides

Paraffin embedded tumors were sliced at 4 µm thickness using HM 340E Electronic Rotary Microtome (Thermofisher Scientific). Tumor slices were rehydrated in xylene and using decreasing concentrations of ethanol. Slices were deparaffinized at low pressure at 100–106 °C for 20 min in citrate buffer at pH 6. PBS buffer with Triton 0.1% was used to permeabilize cells. Unspecific binding was blocked using a PBS buffer with FBS 1%. Slices were incubated for 1 h with anti-human KI67 mouse antibody 556003 (BD Biosciences, Franklin Lakes, NJ, USA) and then with anti-mouse antibody coupled to Alexa 488. DAPI was used to stain nuclei. Images were taken with a Zeiss Axioplan 2 fluorescence microscope coupled to a digital charge-coupled device camera using a 40×/1.3 Plan NeoFluar objective and filter for Alexa 488. The diameters of 20 nuclei of SRC (sample randomly chosen) were measured in two slices of each tumor using imageJ software.

### 2.13. Statistical Analysis

Comparison between H295R and H295R-PM-Ptc+ cells analysis was done using a two tailed student test, * for *p*-value < 0.05, ** for *p*-value < 0.001 and *** for *p*-value < 0.0001.

### 2.14. RNA-Seq

Libraries were generated from 500 ng of total RNA from parental H295R and H295R-PM-Ptc+ cells using Truseq Stranded mRNA kit (Illumina). Libraries were then quantified with Qubit dsDNA High Sensitivity Assay Kit (Invitrogen) and pooled. A measure of 4 nM of this pool was sequenced on a NextSeq 500 platform (Illumina) with 2 × 75 bp paired-end chemistry. All sequencing results were submitted in the GEO database under the super series accession number GSE189424 [28].

Reads were mapped with STAR 2.4.0a on human genome (build hg38). Counting was performed with featureCounts (subread-1.4.6-p1-Linux-x86_64, “--primary-g gene_name -p-s 1-M “ options) using Ensembl GRCh38.76 release. Statistical analysis was performed using R version 3.3.1. The reproducibility of the replicates for each condition was assessed by performing principal component analysis using the R package “htSeqTools” (R package version 1.14.0; data not shown). Functions from the Bioconductor package DESeq2 were used to normalize data for differences in their sequencing depth and assess differential expression. The Benjamini–Hochberg method was used to control the false discovery rate. Results of DESeq2 differential expression analysis are presented in Supplementary Materials Table S1. Genes considered as differentially expressed with a *p*-value < 0.05 and an absolute log2 fold change >1 are listed in Supplementary Materials Table S2. Genes associated with a *p*-value < 0.05 are listed in Supplementary Materials Table S3.

Functional enrichments for Gene Ontology terms, KEGG, WikiPathways and Reactome Pathways were retrieved using Cytoscape [29] and the StringApp plugin [30]. The enrichments of differentially expressed genes reported in Supplementary Materials Table S2 are listed in the “enrichments underexpressed”, “enrichments overexpressed” and “enrichments all” worksheets in Supplementary Materials Table S2.

AMINE (Active Module Identification through Network Embedding) [31] was used to identify modules of genes whose expression was triggered in the biological experiments. The interaction of different genes in the experimental conditions were then compared to a background database, indicating an involvement of these genes in the studied process. String database [32] was used to retrieve gene interaction data with a combined evidence score greater than 0.7. Measurements of the activity of genes are represented by the *p*-values computed by DESeq2.

Internally, the method merges the interaction data with the genes’ associated *p*-values to generate an attributed gene network in which vertices represent genes, edges represent interactions between genes and each vertex is annotated with a numeric attribute reflecting its associated *p*-value. The method relies on Node2vec [33] to learn a vector representation of the interaction network. As a result, each gene is represented by a vector in an embedded space in which the cosine distance between the vectors representing the nodes accurately reflects their proximity in the original network. The algorithm then uses a greedy approach to build increasingly large clusters of genes based on the similarity of their encoding vectors and evaluates them according to a metric considering the activity of the contained genes. The details of the method are described in [31].

However, this whole internal workflow is transparent to users who can simply apply the method on their differential expression data from the web site [34]. On this site, it is possible to perform the identification of the active modules by simply uploading the file generated by DESeq2 and specifying the index of the column containing the gene names, the index of the p-value column and the index of the log2 fold change. The output generated by AMINE consists of an Excel file listing the identified modules.

## 3. Results:

### 3.1. A Small Subpopulation of the ACC Cell Line H295R Presents an Increased Amount of Ptch1 at the Plasma Membrane

**Figure 1:**
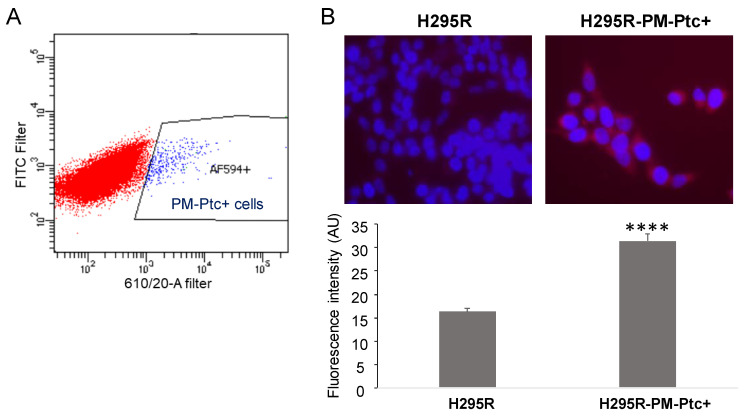
**A small population of ACC cells H295R overexpresses Ptch1 at the plasma membrane**. (**A**) H295R were labeled with an anti-Ptch1 antibody directed against the extracellular loop and cells presenting Ptch1 at their plasma membrane (H295R-PM-Ptc+ AF594+ cells) were sorted. AF594+ in blue represents the percentage of cells with Ptch1 at the cell surface (H295R-PM-Ptc+ cells). (**B**) Surface labeling of Ptch1 using anti-Ptch1 antibody directed against the extracellular loop of Ptch1 (Alexa 594 in red) on nonpermeabilized parental H295R and H295R-PM-Ptc+ cells. Nuclei were stained with DAPI (in blue). The histogram represents the mean ± SEM of Alexa 594 fluorescence intensity per cell (****: *p*-value < 0.00005 (*p*-value = 2 × 10^−36^)).

We previously showed that Ptch1 is well expressed in the ACC cell line H295R [18]. Here, the expression of Ptch1 at the cell surface of H295R cells was addressed by FACS using an antibody directed against an extracellular loop of Ptch1. Surprisingly, only a small percentage (1.26% ± 0.11; *n* = 9) of H295R cells presented Ptch1 at the plasma membrane (PM) (Figure 1A). This subpopulation of H295R cells was sorted and amplified and named H295R-PM-Ptc+. The immunofluorescence study performed with the same anti-Ptch1 antibody on non-permeabilized cells confirmed the presence of higher levels of Ptch1 at the plasma membrane from H295R-PM-Ptc+ cells as compared to parental H295R cells (Figure 1B).

### 3.2. H295R-PM-Ptc+ Cells Are More Resistant to Chemotherapy Than Parental Cells

**Figure 2:**
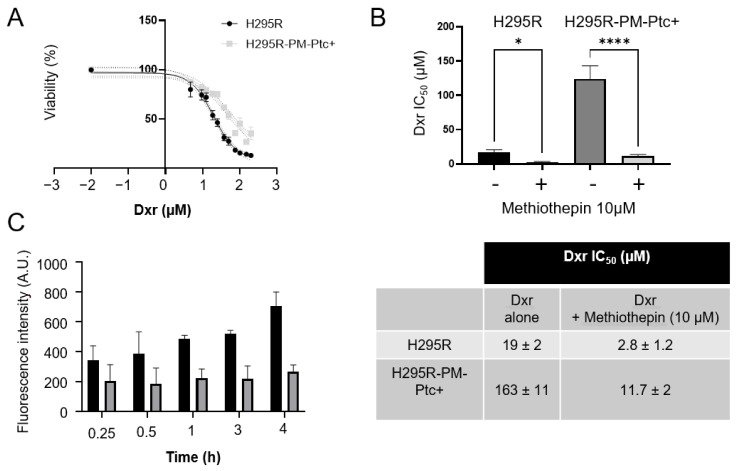
**H295R-PM-Ptc+ cells are more resistant to chemotherapy than parental cells**. (**A**) Doxorubicin (dxr) cytotoxicity. H295R and H295R-PM-Ptc+ cells were treated for 48 h with increasing concentrations of dxr before cell viability measure. (**B**) Doxorubicin IC50 of H295R-PM-Ptc+ and H295R parental cells in the absence or the presence of 10 μM of the Ptch1 efflux inhibitor methiothepin. (**C**) H295R-PM-Ptc+ cells accumulate less doxorubicin than parental H295R cells. Cells on coverslips were incubated with 2 μM dxr for 15, 30, 60, 180 and 240 min and immediately fixed with PFA. Dxr fluorescence was acquired using a filter for Alexa 594 and quantified using ImageJ software. About 100 cells (from three wells) were scored per condition per experiment. All data presented are the mean ± SEM of at least 3 independent experiments. Significance is attained at *p*-value < 0.05 (*), (**** *p* < 0.00005).

When H295R-PM-Ptc+ cells were treated for 48 h with increasing concentrations of doxorubicin (dxr), they showed an increased dxr IC50 compared to parental H295R cells indicating that these cells are more resistant to dxr (Figure 2A,B). This is in good agreement with our previous study showing that H295R cells rendered resistant to dxr express more Ptch1 proteins than control cells [18]. Using the natural fluorescence of dxr, we measured the accumulation of dxr in cells after various incubation times with dxr. As shown in Figure 2C, dxr accumulates less in H295R-PM-Ptc+ cells than in H295R cells. The difference in accumulation increases with the time of incubation with dxr indicating that these cells efflux more dxr than the parental cells. This result is coherent with those obtained in our previous study showing that the depletion by 60% of Ptch1 protein in H295R cells using specific siRNAs equally reduced dxr efflux by 60%, confirming that Ptch1 is a major dxr efflux pump in these cells [18]. To confirm that the increase of resistance to doxorubicin of H295R-PM-Ptc+ cells was related to the increase in the level of expression of Ptch1 at the cell membrane, we treated the cells with methiothepin, an inhibitor of Ptch1 drug efflux activity, in the presence of increasing concentrations of dxr. As shown in Figure 2B, methiothepin increased the cytotoxicity of dxr on H295R-PM-Ptc+ cells even better than on parental H295R cells. Indeed, the presence of 10 µM of methiothepin decreased 14 times and 7 times the IC50 of dxr on H295R-PM-Ptc+ cells and on parental H295R cells, respectively (Figure 2C). This result confirms that the stronger resistance of H295R-PM-Ptc+ cells is correlated to the increased level of Ptch1 at the plasma membrane of these cells.

### 3.3. H295R-PM-Ptc+ Cells Show Superior In Vitro Clonogenic, Migratory and Invasive Capabilities Than Parental H295R Cells

**Figure 3:**
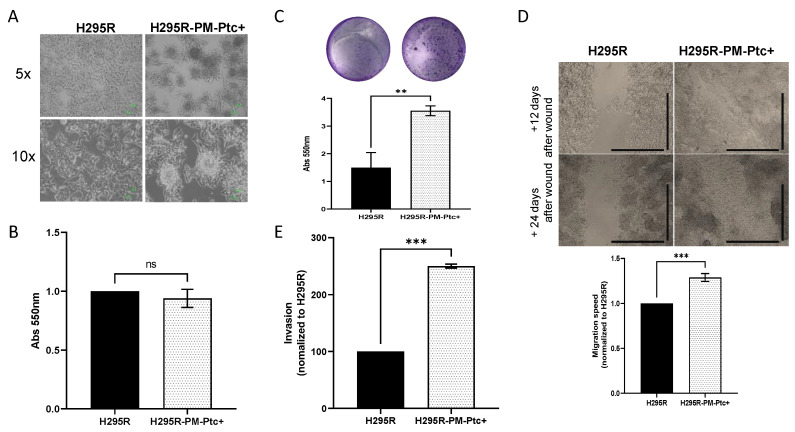
**H295R PM-Ptc+ cells are more aggressive than parental H295R cells**. (**A**) H295R-PM-Ptc+ cells present a different growth pattern. (**B**) H295R-PM-Ptc+ show non-significant lower proliferative properties as H295R cells over 72 h (*n* = 3; no significant difference (ns)). (**C**) H295R-PM-Ptc+ cells form more clones than ACC cells 14 days after seeding 5000 cells/well. Histogram represents the quantification of clones formed using crystal violet for 3 independent experiments (*p* = 0.003). A representative image of clones stained with crystal violet is provided for each cell group. (**D**) H295R-PM-Ptc+ cells migrate faster than H295R cells. Migration ability of the two cell groups was evaluated using wound healing experiment. Slope of migration for 24 h, normalized to H295R, *n* = 5, (*p* = 0.005). H295R-PM-Ptc+ cells completely reclose the wound compared to H295R cells, images taken 12 and 20 days after producing the wound (5× objective, scale = 100 µm). (**E**) H295R-PM-Ptc+ cells are more invasive than parental cells. More H295R-PM-Ptc+ cells were able to pass through Matrigel-coated filters compared to H295R. Invasion was normalized to H295R and proliferation differences, cells passed through the filter were counted and reported to the total cell number (*n* = 2). All data presented are the mean ± SEM of independent experiments. **: *p*-value < 0.005, ***: *p*-value < 0.0005.

We observed that H295R-PM-Ptc+ cells presented a different growth pattern. They formed cell clusters which look like spheroids instead of the single cell monolayer attachment pattern of the parental H295R cells (Figure 3A), and they grew slightly but not significantly slower than parental H295R cells (Figure 3B).

We then compared the ability of each cell group to form clones. For this, H295R-PM-Ptc+ and H295R cells were seeded at 5000 cells/well in 24 well plates for 14 days, and then fixed and colored using violet crystal. The quantification showed that H295R-PM-Ptc+ formed significantly more clones than the parental cell line (Figure 3C).

To compare the migration ability of these cells, we performed a wound healing assay in which, after a scratch in a confluent monolayer of cells, the closing of the wound was monitored by taking a picture of the wound every 15 min for 24 h. To measure the speed of closing, the wound area was measured for each time point. H295R-PM-Ptc+ cells showed a migration speed 1.3 times greater than the parental H295R cell line over the first 24 h (Figure 3D). Accordingly, H295R-PM-Ptc+ cells were able to close the wound completely after 20 days, whereas H295R cells did not.

The invasiveness properties of these cells were addressed using a transwell invasion assay. After correction to take into account the proliferation differences in each experiment, the data showed that the number of cells able to pass through the Matrigel-coated filters was significantly higher with H295R-PM-Ptc+ cells compared to the parental H295R cells (Figure 3E).

Taken together, our results strongly suggest that H295R-PM-Ptc+ cells possess an increased ability to form clones, to migrate and are more invasive than the parental cells.

### 3.4. H295R-PM-Ptc+ Cells Are More Tumorigenic and Metastatic In Vivo Than Parental Cells

**Figure 4:**
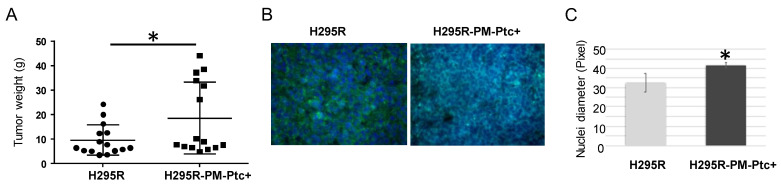
**H295R-PM-Ptc+ cells are more tumorigenic than parental cells in mice**. Each mouse received a renal subcapsular injection of H295R PM-Ptc+ or parental H295R cells (3 × 10^6^ cells; 10 mice for each cell group). After 45 days, mice were sacrificed, tumors were collected, weighed and fixed. (**A**) The mean weight of H295R-PM-Ptc+ tumors is significantly higher than H295R tumors (*n* = 9; *: *p*-value < 0.05 (*p* = 0.018)). (**B**) KI67 immunostaining on tumor slices. KI67 is marked with anti-human KI67 antibody recognized with a secondary antibody coupled to Alexa 488 (green staining) and DAPI (blue staining). Images were taken with an epifluorescence microscope (40×). (**C**) Tumors derived from H295R-PM-Ptc+ cells present bigger nuclei compared to tumors from parental cells. For each tumor, 20 nuclei diameters were measured (SRC-sample randomly chosen) from slides using imageJ software. Six H295R tumors and seven H295R PM-PTC+ tumors (*: *p*-value < 0.005).

Tumorigenicity of H295R-PM-Ptc+ cells was addressed in mice. NMRI nude mice underwent a renal subcapsular injection with H295R and H295R-PM-Ptc+ cells. Forty-five days after the injection, mice were sacrificed, and tumors were collected, weighed, fixed and included in paraffin for immunohistochemistry and immunofluorescence analyses. The results show that H295R-PM-Ptc+ cells formed tumors significantly bigger compared to the parental H295R cells (Figure 4A). The labelling of tumor slices with an antibody against Ki67, a marker of proliferative cells, showed no difference in the amount of Ki67-positive cells between both tumor groups (Figure 4B). Interestingly, the nuclei in H295R-PM-Ptc+ cells were significantly bigger when compared to those of the parental cells (Figure 4C).

**Figure 5:**
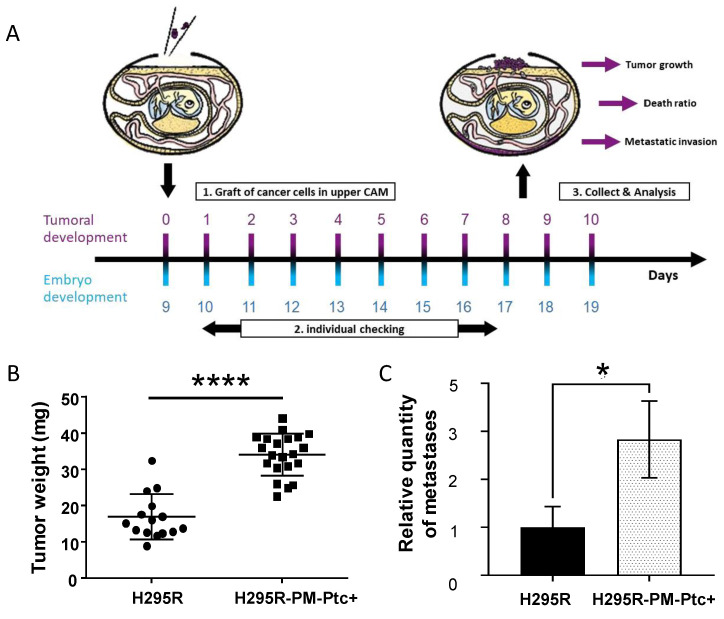
**H295R-PM-Ptc+ cells are more tumorigenic and metastatic than parental ACC cells in ovo**. (**A**) Schematic representation of the study. On day 9 of egg development (E9) 3 × 10^6^ H295R-PM-Ptc+ and H295R cells were injected in the chorioallantoic membrane (CAM) from two groups of 20 eggs. At E18 tumors from CAM and embryos were collected. (**B**) H295R-PM-Ptc tumors collected on E18 were bigger than H295R tumors (*n* = 15 for H295R graft and *n* = 21 for H295R-PM-Ptc+; ****: *p*-value < 0.00005). (**C**) Metastasis in liver was addressed using human Alu sequences by real time qPCR. H295R-PM-Ptc+ xenografted cells form more metastases in the embryo liver compared to H295R xenografted cells (mean ± SEM *n* = 9, *: *p*-value < 0.05 (*p* = 0.03)).

Tumorigenesis and metastases were then addressed in ovo. This system consists of the injection of H295R-PM-Ptc+ cells or parental H295R cells at the chorioallantoic membrane (CAM) of two groups of fertilized white leghorn eggs at day 9 of embryonic development (E9). At day 18 of embryonic development (E18), tumors and embryos were collected and analyzed (Figure 5A). The tumors originated from H295R-PM-Ptc+ were significantly bigger than H295R tumors, confirming the results obtained in mice (Figure 5B). Metastasis quantification was addressed using real time qPCR with human ALU sequences allowing the detection of the presence of human cells in the chicken embryos’ organs. As ACC cells have a hepatic tropism, genomic DNA was extracted from the livers of the embryos. Real time qPCR revealed that there were more metastases in the livers of embryos from eggs injected with H295R-PM-Ptc+ compared to those from eggs injected with parental H295R cells (Figure 5C).

### 3.5. H295R-PM-Ptc+ Cells Differentially Express Genes Associated with EMC, Invasion, Metastasis and Cancer Stem Cell Properties

Differential gene expression analysis with DESeq2 was used to estimate the changes between the experimental conditions. Standard filtering with a *p*-value < 0.05 and an absolute log2 fold change >1 holds 57 underexpressed genes and 15 overexpressed genes (Supplementary Materials Table S2). Functional enrichments for Gene Ontology terms, KEGG, WikiPathways and Reactome Pathways identified with a false discovery rate (FDR) <0.05 are listed in the “enrichments underexpressed”, “enrichments overexpressed” and “enrichments all” worksheets in Supplementary Materials Table S2. No enrichments could be attributed to overexpressed genes. Underexpressed genes, as well as the set of all differentially expressed genes (DEG) were enriched with fairly broad annotations related to development, morphogenesis, cell adhesion, response to stimulus or extracellular matrix organization. Being more stringent, or looser in the selection of genes that are considered over- or underexpressed does not help to obtain more precise insight into the biological processes that are at work.

**Figure 6:**
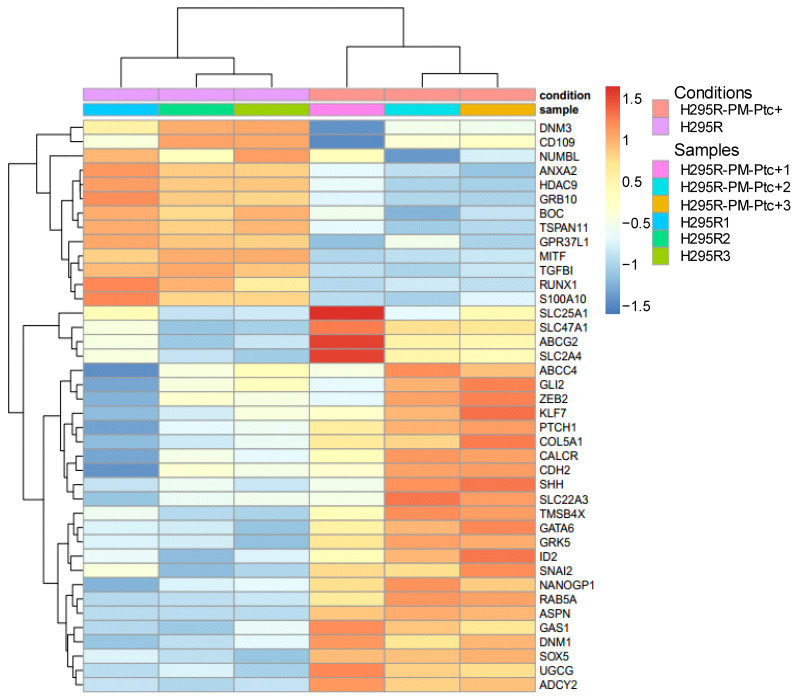
**Heat map of differentially expressed genes (DEG) between H295R-PM-Ptc+ and parental H295R cells of interest for their role in cancer**.

By selecting genes with a *p*-value < 0.05 and setting no constraints on their minimum variation, we obtained 2374 differentially expressed genes with 1076 overexpressed genes and 1298 underexpressed genes (Supplementary Materials Table S3). The functional enrichments performed on this extended list result in annotations that were similar and as broad as those obtained with the more stringent list presented in Supplementary Materials Table S2. From the 2374 DEGs, we selected 41 DEGs of interest regarding the characteristics of H295R-PM-Ptc+ cells with 26 overexpressed (in red) and 15 underexpressed (in blue) (Table 1, Figure 6). As expected, Ptch1 gene was one of the most overexpressed genes. All these DEGs are related to tumorigenicity, playing a role in the Hedgehog signaling activation, tumor progression, cancer stem cell (CSC) maintenance, chemotherapy resistance, epithelial to mesenchymal transition (EMT), metastasis and cancer cell survival or endocytosis.

**Figure 7:**
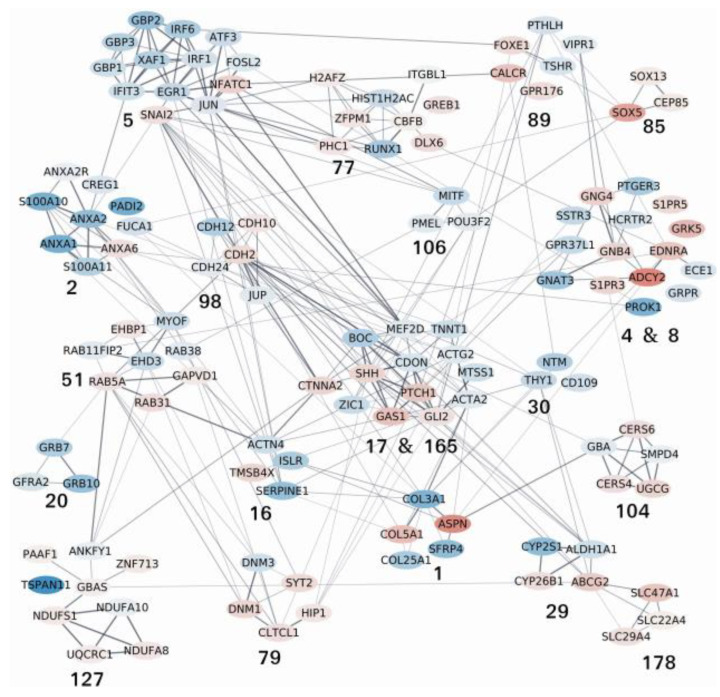
**Network of protein–protein interactions between the members of the active modules containing one or more of the identified genes of interest listed in **Table 1. The nodes on the network correspond to genes. Node colors represent the log2 fold change values of the corresponding gene on a scale varying from blue (for the most underexpressed genes) to red (for the most overexpressed genes). Edges correspond to interactions reported in the String database with a combined evidence score ≥0.4. Each module is identified by its number (specified in Supplementary MaterialsTables S2 and S3). The complete lists of the enrichments of all the modules are shown in Supplementary Materials Table S4.

From the raw output of DESeq2, we performed a search for active modules using AMINE [31] which is a method designed to identify the modules of genes that are triggered in a biological experiment (the output of AMINE is presented in Supplementary Materials Table S4). Among the 188 active modules identified by AMINE, we selected those that contain one or more of the identified genes of interest listed in Table 1. This enables the identification of 21 modules, whose composition is detailed in Supplementary Materials Table S5. A graphical representation of the interactions between the proteins encoded by the genes within the outlined modules is proposed in Figure 7. In the figure, each module is identified by its number (specified in Supplementary Materials Table S5). The details of all the enrichments of the modules with a FDR < 0.05 are presented in Supplementary Materials Table S6. Some modules that exhibit many interactions between their members have been grouped together; this is the case for modules 4 and 8 as well as 17 and 165. Unlike the enrichments obtained by selecting only the most deregulated genes, the identification of active modules makes it possible to identify more specific molecular pathways or processes. Modules 17 and 165 are in the center of the graph and interact with many of the other modules. These modules contain components of the Hedgehog signaling pathway, and their differential expression reveals an activation of the pathway. Indeed, Shh and Gli2 which are well-known activators of the pathway and Ptch1, which is an Hh target gene, are overexpressed. CDON and BOC are essential cell surface modulators of the Hh pathway. In their absence, Gas1, an Hh-binding protein, mediates Hh signaling [35]. The number and composition of the 21 active modules, their representative enrichment and the role of differentially expressed genes (DEGs) in cancers are summarized in Table 2.

**Figure 8:**
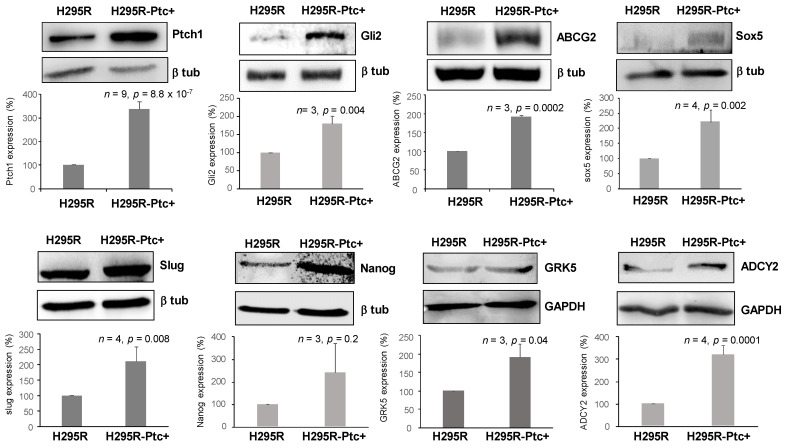
**Protein expression of some differentially expressed genes between H295R cells parental and H295R PM-Ptc+ cells FACS sorted and amplified**. Western blots were performed using anti-DEG antibodies, and β-tubulin or GAPDH antibodies for the loading control. Signals on Western blots were quantified using ImageJ software. Data presented are the mean ± SEM of at least 3 independent experiments. *p*-values were calculated using Student’s t-test.

We then performed Western-blots on H295R and H295R-PM-Ptc+ cell extracts to find out if the differences observed at the RNA level were reflected at the protein level for some DEGs. Figure 8 shows that the protein level of several DEGs such as Ptch1, Gli2, ABCG2, SOX5, GRK5, ADCY2, Nanog and SNAI2 (Slug) was significantly increased in H295R-PM-Ptc+ cell extracts. This result is in good agreement with RNAseq data and shows that some proteins involved in cancer stem cell maintenance are overexpressed in H295R-PM-Ptc+ cells.

## 4. Discussion

In this study, we isolated and characterized a small subpopulation of the adrenocortical carcinoma (ACC) cell line H295R that overexpresses Ptch1 and presents more Ptch1 at their plasma membrane, called H295R-PM-Ptc+ (Figure 1). This cell subpopulation was found to be more resistant to the chemotherapeutic drug doxorubicin (Figure 2). This is in good agreement with our previous study showing that H295R cells rendered resistant to doxorubicin express more Ptch1 than control cells [18]. These results also strengthen our previous data suggesting that the Hedgehog receptor Ptch1 strongly contributes to chemotherapy resistance of H295R cells by exporting drugs such as doxorubicin out of these cells [18]. We were more surprised by the results showing that this subpopulation of cells grew as spheroids, and had a greater capability of clonogenicity, migration and invasion in vitro than the parental cells (Figure 3). Moreover, xenograft experiments performed in mice and in ovo demonstrated that cells amplified from the subpopulation of H295R-PM-Ptc+ were more tumorigenic and more metastatic than the parental cells in vivo (Figure 4 and Figure 5). This is in good agreement with in vitro observations, and strongly suggests that H295R-PM-Ptc+ have properties similar to those of cancer stem cells or tumor-initiating cells or persistent cells. Cancer stem cells (CSCs) or persistent cells are a subpopulation of cells identified in most types of liquid and solid cancers by cell surface markers more or less specific of the tumor-type that have the driving force of carcinogenesis [36,37]. CSCs present a different behavior when compared to the other cells within the same tumor, in particular, they have distinctive self-renewal, proliferation and differentiation faculties. CSCs play a critical role in cancer initiation, progression and recurrence, metastases formation, and resistance to therapy. The acquisition of this aggressive and MDR phenotype is due to different cellular mechanisms, i.e., drug-efflux pump activation, enhanced capacity of DNA damage repair, dysregulation of signaling pathways involved in cell growth and development, altered cell metabolism and diminished apoptosis response.

To better characterize H295R-PM-Ptc+ cells and confirm the hypothesis that these cells are cancer stem-like cells, we performed an RNA-seq analysis which revealed that 2374 genes were significantly differentially expressed in H295R-PM-Ptc+ derived cells in comparison with H295R parental cells. The overexpression of some of these genes was confirmed at the protein level by comparing H295R-PM-Ptc+ cell extracts to parental H295R cell extracts (Figure 8). These RNA-seq and the Western-blots analyses confirmed the strong overexpression of Ptch1 in H295R-PM-Ptc+. Indeed, a variety of primary tumors and cancer cell lines (i.e., lung, ovary, prostate, colon, brain and myeloid leukemia) overexpress Ptch1 (see the Human Protein Atlas website (http://www.proteinatlas.org/ENSG00000185920-PTCH1/cancer, [14] for a review, accessed on 10 January 2022). Im and colleagues showed that in 190 over 334 tissue microarrays from breast cancer patient samples the overexpression of Ptch1 was significantly correlated with a more aggressive tumor growth, advanced cancer stages and lymph node metastasis [38]. Papadopaulos and co-workers reported an overexpression of Ptch1 in colorectal cancers, and their analysis of esophageal biopsy specimens from patients treated with chemotherapy revealed elevated levels of Ptch1 expression in 76% of the cases [39]. Interestingly, decreased response to chemotherapy, large tumor size and locoregional progression of esophageal squamous cell carcinoma seem associated to a high expression of Ptch1 [40]. Furthermore, Ptch1 expression has been proposed to be an early marker for gastric and thyroid cancers [15,16], and more recently a prognosis marker for relapse in high-risk prostate cancer patients [41]. Moreover, our recent studies showed that Ptch1 is present in primary tumor samples from all adrenocortical carcinoma patients of the cohort studied [18], and that Ptch1 is present in the metastases of all 365 melanoma patients of a TCGA cohort and correlates with a poorer the prognosis [19].

The differentially expressed genes (DEGs) from the RNAseq analysis have been grouped into 188 active modules. Twenty-one of these modules and their interactions are presented in the Figure 7 and the Table 2. The differential expression of genes from modules 17 and 165 revealed an activation of the Hedgehog (Hh) signaling pathway in good agreement with the overexpression of **Ptch1** which is an Hh target gene. Moreover, the overexpression of the transcription factor **Gli2** involved in the Hh signaling activation was also shown at the protein level (Figure 8). The Hh signaling is a determinant pathway for tumor progression and cancer stem cell maintenance. For example, decreased **CDON** expression was also observed in a large fraction of human colorectal cancer and was associated with intestinal tumor progression in mice [42]. **BOC** inactivation resulted in reduced proliferation and progression of early medulloblastomas to advanced cancer [43]. Interestingly, CDON and BOC have been suggested to regulate Hh signaling through the modulation of Ptch1 distribution at the cell surface. Their down-expression in H295R-PM-Ptc+ cells could explain the presence of more Ptch1 at the plasma membrane of these cells [44]. Remarkably, modules 17 and 165 are in the center of the graph and interact with many of the other modules. The differential expression of the genes contained in modules 1, 4 and 8, 5, 16, 17 and 165, 51, 77, 79, 98 and 178 is associated with epithelial to mesenchymal transition (EMT) which can be triggered by tumor cells, cancer development and progression, metastasis, and correlated with poor prognosis, while the differential expression of the genes contained in modules 1, 5, 17 and 165, 20, 29, 85, 89 and 106 is more involved in CSC maintenance, and that of the genes contained in modules 5, 17 and 165, 29 and 104 is associated with chemotherapy resistance. Some illustrations are given below:

Proteins from **module 98**, and more particularly the N-cadherin **CDH2**, present strong interactions with proteins from modules 17 and 165. Cadherins are transmembrane glycoproteins involved in cell–cell adhesion during embryogenesis, tissue morphogenesis, differentiation and carcinogenesis. The loss of epithelial cadherin (E-cadherin), by affecting cell–cell adhesion, induces EMT and tumor progression. Many signaling pathways activated during tumorigenesis affect cadherin cell–cell adhesion which contributes to tumor progression and metastasis [45]. N-cadherin promotes thyroid tumorigenesis through modulating major signaling pathways [46]. CDH2 has been reported to be highly expressed in metastatic liver cancer. By analyzing gastric cancer (GC) patients in two independent cohorts, Luo and co-workers showed that cadherins CDH2, CDH6, CDH7 and CDH10 were significantly associated with a poor GC prognosis [47]. Moreover, the knockdown of **JUP**, a cell–cell junction protein homologue of β-catenin involved in adhesion junction and desmosome composition, causes EMT, promotes GC-cell migration and invasion, and was closely correlated with GC malignancy and poor prognostics [48].

Proteins from **module 5**, and more specifically **Snai2** (Slug), are strongly interacting with proteins from modules 17 and 165 and 98. H295R-PM-Ptc+ cells present an overexpression of Snail2 at the RNA and protein level. This C2H2-type zinc finger transcription factor also called Slug has antiapoptotic activity, and participates in EMT, tumor progression, stem and or/progenitor maintenance, tumor metastasis, cellular differentiation, vascular remodeling, and DNA damage repair. Snai2 plays a role in breast carcinoma as well as leukemia by downregulation of E-cadherin, which supports the mesenchymal phenotype and enables metastasis of tumor cells [49]. Snai2 is also associated with a poor prognosis of luminal B HER2^+^/ERBB2^+^ breast cancers [50] and directly contributes to cisplatin resistance in ovarian cancer [51]. **NFATC1**, a nuclear factor of activated T-cells c1, is also upregulated in H295R-PM-Ptc+ cells and is associated with malignancy in several cancer models. Different NFAT isoforms are overexpressed in diverse hematologic malignancies and solid tumors. The overexpression of this gene seems to be involved in single cell fate, increasing the ability of the cancer cells to migrate/invade and differentiate but also to survive in both the tumor and the associated microenvironment. NFATC1 overexpression in high-grade serous ovarian carcinomas was associated with poor overall survival and of early relapse [52]. **GBP1, 2, 3** and **ATF3** genes are downregulated in H295R-PM-Ptc+ cells. Guanylate-binding proteins belongs to the dynamin superfamily. These GTPases are important effectors of cell dynamics acting on membrane, cytoskeleton and cell cycle progression. GBP1, considered a tumor-repressor gene, was found to be downregulated in colorectal cancer [53]. It was reported that transfection of GBP2 in colorectal cancer (CRC) cells inhibited their growth and increased their sensitivity to paclitaxel in a paclitaxel-resistant CRC, impairing Wnt signaling [54]. Dysregulation of ATF3, a cyclic AMP-dependent transcription factor, has been observed in diverse cancers, especially in various step of tumorigenesis. Low ATF3 expression was correlated to shorter survival and poorer prognosis in gastric cancer patients [55]. Moreover, the expression of ATF3 in SW620 CRC cells inhibits cell growth and stem cell-like characteristics [56]. Overall, differential expression of genes from module 5 are involved in the maintenance of the mesenchymal phenotype, invasive migration, metastasis, poor prognosis, and chemotherapy resistance.

**Module 1** is in interaction with modules 17 and 165 and 16. It contains five DEGs with two being highly overexpressed in H295R-PM-Ptc+ cells: **ASPN** and **COL5A1**. The collagen family gene **COL5A1** has been identified as a possible predictor of recurrence after radiation therapy for vestibular schwannoma (VS) [57] and related to brain metastasis in breast cancer patients [58]. The extracellular matrix protein asporin (**ASPN**) has been shown to promote cell migration and invasion [59] and may be a key molecule in facilitating tumor spreading [60]. Sasaki and co-workers recently suggested that asporin expression could reprogram cancer cells to acquire resistance to oxidative stress [61].

Proteins from **module 16** are in interaction with those from modules 17 and 165, 98 and 1. **TMSB4X** (Thymosin beta 4 X-linked), which is upregulated in H295R-PM-Ptc+ cells, has been proposed to suppress E-cadherin expression, and to promote cancer cell growth and migration. The overexpression of TMSB4X was found significantly associated with poor prognosis of overall survival and recurrence-free survival in head and neck squamous cell carcinoma (HNSCC) patients. The global proteomics analysis identified TMSB4X as a new biomarker of HNSCC whose functions resulted in enhanced proliferation and metastasis in vitro and in vivo [62].

**Modules 4 and 8** contain the most overexpressed gene, ADCY2. **ADCY2** overexpression was confirmed at the protein level. The adenylate cyclase ADCY2 is overexpressed in prostate and colon cancer metastases, and in pancreatic neuroendocrine tumors [63]. It is considered as a strong metastatic marker. The sphingosine-1-phosphate receptor 3 (**S1PR3**) and 5 (**S1PR5**) are closely related G-Protein-coupled receptors involved in the lipid-mediated regulation of CSC via Notch signaling and cancer cell survival [64,65]. The binding of the lipid S1P (Sphingosine-1-phosphate) to the Sphingosine-1-phosphate receptors (S1PR1, S1PR2, S1PR3, S1PR4 and S1PR5) triggers different pathways involved in cell differentiation, proliferation, angiogenesis and migration [66]. Upregulation of the guanine nucleotide binding-protein gamma subunit 4 protein (**GNB4**) was significantly associated with primary tumor, nodal metastasis, histological grade, vascular invasion and mitotic rate [67,68]. High expression of **EDNRA** is associated with metastasis and poor outcome in patients with advanced bladder cancer [69]. **PROK1** gene which is involved in cell-to-cell contact, epithelial tissue differentiation, Ca2+ release, lipid synthesis, and chemotaxis is downregulated in H295R-PM-Ptc+ cells. Prostaglandin receptor EP3 (**PTGER3**) downregulation was shown to contribute to prostate carcinogenesis and to progression from androgen-dependent prostate cancer to castration-resistant prostate cancer [70].

In **Module 79**, dynamin 3 (**DNM3**) which functions as a tumor suppressor in various malignancies is downregulated in H295R-PM-Ptc+ cells. The low expression of DNM3 was significantly associated with high pathological grading of cervical cancer [71]. In contrast, **DNM1** is overexpressed in H295R-PM-Ptc+ cells, as in colon cancer where high DNM1 expression was significantly correlated with perineural and lymphatic invasion and predicted poor prognosis [72]. Moreover, the overexpression of Huntingtin-interacting protein 1 (**HIP1**) has also been observed in prostate and colon tumor cells where HIP1 expression was significantly associated with prostate cancer progression and metastasis. Studies suggest that HIP1 is a cellular survival factor which may play a role in tumorigenesis by allowing the survival of precancerous or cancerous cells [73].

The overexpression of genes from **module 104** is associated with sphingolipid pathway, migration, metastasis and chemotherapy resistance. Sphingolipids are lipids associated to the membrane implicated in signaling pathways which regulate cell migration, growth and death. In cancers, sphingolipids regulate pathways involved in tumor progression, metastasis, invasion and lethal mitophagy [74]. The activity of **UGCG** (UDP-glucose ceramide glucosyltransferase) is related to multidrug resistance and cell proliferation in different cancer types. In breast cancer cells, the overexpression of UGCG was shown to increase glycolysis and oxidative phosphorylation [75]. The ceramide synthase encoded by the gene **CERS6** was shown to be required for cell migration and metastasis in lung cancer [76].

**Module 29** contains four DEGs in strong interaction with modules 17 and 165. The RNAseq showed that **ABCG2** is overexpressed in H295R-PM-Ptc+ cells, and this was confirmed at the protein level by Western-blot. This ABC transporter is a direct transcriptional target of Hh signaling and has been shown to be involved in drug tolerance [77]. ABCG2 is a well-known marker of cancer stem-like cells and contributes to the resistance of these cells to chemotherapy [36].

**Module 2** contains the two most underexpressed genes **S100A10** and **ANXA2**. A decreased expression of the S100 calcium-binding protein (**S100A10**) has been shown to reduce the intracellular calcium concentration and the rate of phagocytosis. It may be associated with an undifferentiated phenotype and poor prognosis in gastric cancer [78]. Annexin 2 (**ANXA2**) is an important regulator of cell–cell adhesion. This protein is negatively correlated with the differentiation status of ESCC tumors with less differentiated malignant tumors, having the lowest ANXA2 levels. ANXA2 depletion has been shown to cause the depletion of S100A10 protein [79]. Indeed, S100A10 associates with ANXA2 and ANXA1 in heterotetramers involved in the regulation of endocytosis, exocytosis, focal adhesion dynamics, cell proliferation, oxidative stress and apoptosis [80,81]. Silencing ANXA2 was shown to downregulate S100A10 and to inhibit breast cancer proliferation and invasion [82]. Moreover, a tissue microarray analysis described that decreased expression of ANXA1 is correlated with breast cancer development and progression [83].

Genes from **module 51** are also involved in membrane trafficking, endocytosis, cancer cell motility and invasiveness, cancer development and progression and metastases. Two genes coding for RAB proteins are overexpressed in H295R-PM-Ptc+ cells, **RAB5a** and **RAB31**. The RAB protein family belongs to the subgroup of the GTPase superfamily. RAB proteins participate in cellular trafficking by regulating the dynamics of the membrane compartments, the Golgi complex organization and the sorting and delivery of secretory and membrane proteins. There is now a special focus on members of the RAB family due to the possible implication in cancer progression [84]. RAB5a overexpression has been identified to be involved in cancer cell motility and invasiveness. It has been associated with lung, stomach, and hepatocellular carcinomas [85], and with axillary lymph node metastasis in breast cancer patients [86]. Increased RAB31 expression in cancer-associated fibroblasts was suggested to promote colon cancer progression [87].

**Module 77** contains genes whose differential expression is associated with negative regulation of differentiation. **RUNX1** (runt-related transcription factor 1) is known as a tumor suppressor in hematopoietic malignancies. Low RUNX1 expression is associated with poor patient survival [88]. In renal cell carcinoma, **DLX6-AS1** (long non-coding RNA (LncRNA) distal-less homeobox 6 antisense 1) was shown as an oncogene, and its expression was positively correlated with the development and progression of the tumor [89]. DLX6-AS1 promotes cell proliferation, migration and EMT of gastric cancer [90], and enhanced secondary cisplatin resistance of lung squamous cell carcinoma [91]. **GREB1** (an estrogen receptor-regulated tumor promoter) overexpression in ovarian cancer cell lines increased cell proliferation and migration and promoted a mesenchymal morphology [92]. 

The overexpression of genes from **modules 85 and 89** is associated with CSC maintenance, EMT, invasion and metastasis. The transcription factors SOX, belonging to the sex-determining region Y (SRY)-related HMG-box family, are important for cell fate determination, embryonic and cancer development. RNAseq and Western-blot data indicate that **SOX5** is overexpressed in H295R-PM-Ptc+ cells compared to parental H295R cells. SOX5 is known to participate in EMT in different cancer types (i.e., breast, prostate, hepatocellular, lung adenocarcinoma and osteosarcoma) [93,94,95]. **SOX13** promotes colorectal cancer metastasis by transactivating SNAI2 and c-MET [96] and regulates cancer stem-like properties and tumorigenicity in hepatocellular carcinoma cells [97]. Calcitonin receptor **CALCR** is a G protein-coupled receptor that binds the peptide hormone calcitonin and is involved in the maintenance of calcium homeostasis. Its overexpression has been shown to keep muscle stem cells in a quiescent state [98]. The forkhead box (Fox) family of transcription factors consists of more than 50 proteins that are Hh signaling targets and involved in tissue-specific transcription and cell fate determination during embryogenesis and cell survival. **FOXP1** was shown to control mesenchymal stem cell commitment and senescence during skeletal aging [99].

Interestingly, the downregulation of two genes from **module 106** are associated with cancer cell phenotype switching: the microphthalmia-associated transcription factor (**MITF**) and the POU domain transcription factor **POU3F2** (better known as BRN2) [100]. Cells with low MITF have been assigned a quiescent invasive phenotype and display invasive properties [101]. Moreover, a low transcriptional activity of MITF would predict poor outcomes for melanoma patients. A zebrafish model that mimics human resistant-melanoma subtypes exhibiting low MITF showed an upregulation of genes involved in stemness and invasiveness [102]. Goding and co-workers reported that the level of MITF induces different states of melanocytes: maintenance of differentiation (MITF-high), proliferation (MITF-intermediate) or slow-proliferative and invasive cells with tumor-initiating properties (MITF-low). Melanoma cells could therefore change reversibly from a proliferative status to an invasive state according to the so-called rheostat model [103].

Therefore, the results from our RNA-seq study are in good agreement with the features of H295R-PM-Ptc+ cells observed in experiments reported in the Figure 1, Figure 2, Figure 3, Figure 4 and Figure 5, and reveal several aspects that help us to better understand and define H295R-PM-Ptc+ cells:

1. The differential expression of genes from several modules suggests an inhibition of membrane trafficking and endocytosis. Moreover, gene coding for **ST6GAL1** and **CERS6** known to inhibit the clathrin-independent endocytosis mediated by glycosphingolipid and lectins (GL-Lect) [104] are overexpressed in H295R-PM-Ptc+ cells. The differential expression of these genes could inhibit the endocytosis of Ptch1 normally induced by its ligand Sonic hedgehog and explain the presence of Ptch1 at the plasma membrane of H295R-PM-Ptc+ cells.

2. The overexpression of Ptch1 and its presence at the plasma membrane of H295R-PM-Ptc+ cells explains that these cells are more resistant to chemotherapy. Several other genes known to induce chemotherapy resistance are also overexpressed, supporting the chemoresistance feature of these cells.

3. The differential expressions of genes from almost all the modules are involved in cancer progression, cell migration or invasion, and are often associated with poor prognosis.

All these characteristics suggest that these cells are cancer stem-like or persistent cells. Indeed, several differentially expressed genes are known to be involved in stem cell and/or quiescence maintenance. The differential expression of other genes has been associated to de-differentiation in different tumors. Several genes specific to adrenocortical carcinoma are downregulated. One of these genes is **STAR** that encodes for a steroidogenic acute regulatory protein responsible for cholesterol transport to the mitochondria which is the rate-limiting step in steroid hormone production [105]. This transport protein is present in steroid-producing cells such as ovary theca and luteal cells, testis Leydig cells and some adrenocortical cells. A lower expression of steroidogenic enzymes such as STAR is consistent with a less differentiated phenotype. Synaptophysin (**SYP**) is an integral membrane glycoprotein that occurs in presynaptic vesicles of neurons and in similar vesicles of the adrenal medulla. A study from Wiedenmann and co-authors [106] concluded that synaptophysin was expressed independently of other neuronal differentiation markers and proposed that it be used as a differentiation marker in tumor diagnosis. ***ZNRF3*** was the most frequently altered gene, corresponding to 21% of ACC cases [107]. This gene encodes a cell surface transmembrane E3 ubiquitin ligase that acts as a negative feedback regulator of the canonical Wnt/β-catenin signaling. The study performed on a cohort of 82 adults with ACC suggested that the low expression of ZNRF3 was associated with a decrease in overall survival, while high ZNRF3 expression was associated with optimistic recurrence-free survival and concluded that low expression of ZNRF3 is a negative prognostic marker of ACC [108]. Accumulating evidence indicates that H295R-PM-Ptc+ cells have a de-differentiated state.

Two melanoma cell subpopulations were described as contributing to targeted therapy and immunotherapy resistance [109]. These two subpopulations exhibited a slow cell cycle activity, a de-differentiated state and invasiveness, and were described by two different models, namely, the cancer stem cell (CSC) model and the microphthalmia-associated transcription factor (MITF)-rheostat phenotype switching model. In the CSC model, melanoma cells are organized hierarchically. Cells can differentiate from CSCs to progenitor cells and then to fully differentiated melanoma cells but cannot de-differentiate in the opposite direction. CSCs contribute to cell survival and multidrug resistance. They can give rise to melanoma cell populations more resistant to treatments. In the MITF-rheostat model, melanoma cells are organized horizontally. Their proliferative (high levels of MITF expression (MITF-high) or invasive (low levels of MITF (MITF-low) phenotypes are interchangeable. In this model, therapeutic resistance is induced by senescent subclones exhibiting extremely high or low MITF expression levels. Bai and co-workers [109] proposed a new model explaining the development of therapeutic resistance by the dynamic fluctuation of cell states providing a reservoir of cells for tumor reorganization. This model combines cell state dynamic oscillation at the single-cell level with the cell ensemble continuous reshaping at the population level. This could also be the case of the ACC H295R cell line and explain the presence of a small population of H295R cells with stemness, invasive and chemoresistance properties.

Altogether, our study strongly supports the hypothesis that H295R-PM-Ptc+ subpopulation has cancer stem-like or persistent cell properties. If present in ACC patients, these cells could be responsible for therapy resistance, relapse and metastases, and may be eliminated by using a Ptch1 drug efflux inhibitor in combination with chemotherapy. Moreover, the presence of Ptch1 at the cell surface could be a marker of the presence of these persistent cell populations in ACC patient biopsies.

## Figures and Tables

**Table 1:** Differentially expressed genes (DEG) between H295R-PM-Ptc+ and parental H295R cells selected for their role in cancer. Genes overexpressed are indicated in red and genes underexpressed are in blue.

GDE	H295R-PMPtc+/H295R Log2	*p*-Value	Role in Cancer
**Hedgehog (Hh) Signaling Pathway**			
PTCH1	+0.629	1.08 × 10^−6^	Hh target gene. drug efflux. chemotherapy resistance
GAS1	+0.724	4.52 × 10^−8^	Hh pathway activation
SHH	+0.421	2.82 × 10^−3^	
BOC	−0.794	3.25 × 10^−7^	BOC endocytosis via NUMB is required for Ptch1 internalization and Shh signaling
HDAC9	−0.925	6.59 × 10^−17^	Negative regulator of Hh signaling
GPR37L1	−0.548	1.27 × 10^−4^	Interacts with Ptch1 for Hh signaling modulation
NUMBL	−0.286	1.47 × 10^−2^	Negative regulator of canonical Shh signaling. Required for BOC and Ptch1 internalization
**Tumor Suppressor**			
DNM3	−0.427	1.21 × 10^−4^	Decreased expression is associated with worse prognosis
RUNX1	−0.898	9.00 × 10^−11^	Represses breast CSC phenotype through direct inhibition of Zeb1/2. Low RunX1 expression is associated with poor patient survival
**CSC Maintenance**			
UGCG	+0.418	2.70 × 10^−7^	CSC maintenance and chemotherapy resistance. increase antiapoptotic gene expression
SOX5	+1.149	7.70 × 10^−17^	Overexpressed in CSC in lung cancer, promotes migration, invasion and metastasis. Predicts poor prognosis
NANOGP1	+0.424	6.42 × 10^−4^	Self-renewal transcription factor, maintenance of CSC, EMT and metastasis.
GATA6	+0.444	1.18 × 10^−4^	Maintenance of stem cell phenotype, regulated by Nanog
KLF7	+0.456	9.06 × 10^−4^	Upregulated in quiescent cells Restores hematopoietic stem cells niche
COL5A1	+0.754	4.58 × 10^−9^	Quiescence and self-renewal of stem cells
CALCR	+0.696	1.90 10^−6^	Maintains cells in a quiescent state
MITF	−0.564	1.58 × 10^−10^	Low MITF is associated to slow cycling and senescence phenotype, dedifferentiation and treatment resistance
GRB10	−1.104	5.20 × 10^−16^	Deletion promotes hematopoietic stem cell self-renewal and regeneration
**CSC and Chemotherapy Resistance**			
ABCG2	+0.592	1.39 × 10^−4^	Drug efflux. Resistance to chemotherapy
ABCC4	+0.352	1.94 × 10^−2^	Chemotherapy resistance. CSC marker
SLC47A1	+0.667	6.46 × 10^−8^	SLC transporters in chemotherapy resistance
SLC2A4	+0.429	9.32 × 10^−4^	Energy production of cancer cells, migration, metastasis
SLC22A3	+0.339	4.10 × 10^−3^	Cell invasion and filopodia formation, metastasis
ID2	+0.294	1.08 × 10^−3^	Inhibition of differentiation and maintenance of self-renewal and multipotency in stem cells
**EMT**			
TMSB4X	+0.434	6.13 × 10^−5^	Suppresses E-cadherin expression, promotes cancer cell growth and migration
SNAI2	+0.295	1.36 × 10^−3^	Triggers EMT, blocks cell cycle and confers resistance to cell death
RAB5A	+0.256	8.33 × 10^−4^	Migration and metastasis
ASPN	+1.476	2.51 × 10^−22^	Promotes cell migration and invasion
CDH2	+0.390	2.63 × 10^−3^	Mesenchymal marker
CD109	−0.425	6.50 × 10^−3^	Co-receptor of TGFB1 Inversely correlates with EMT, migration and invasion
TGFBI	−1.75	2.83 × 10^−29^	TGFB1 deficiency predisposes mice to tumor development
**Metastasis, Cancer Cell Survival**			
ADCY2	+1.56	5.63 × 10^−24^	Involved in metastasis. High expression related to worse survival.
DNM1	+0.452	1.05 × 10^−4^	Invasion and metastasis
GRK5	+0.831	3.84 × 10^−9^	Tumor progression
**Endocytosis**			
S100A10	−1.473	3.86 × 10^−21^	Undifferentiated phenotype and poor prognostic-low level in metastatic melanoma
ANXA2	−1.144	8.08 × 10^−16^	ANXA2 and S100A10 heterotetramer is involved in regulation of endocytosis.
ST6GAL1	+0.668	1.54 × 10^−10^	Inhibition of endocytosis GL-Lect dependent. High tumor grade, metastasis and reduced patient prognosis
CERS6	+0.314	5.55 × 10^−3^	
**Adrenocortical Carcinoma Markers**			
STAR	−0.289	4.21 × 10^−2^	Lower expression this steroidogenic enzymes is associated with a less differentiated phenotype.
ZNRF3	−0.315	3.21 × 10^−3^	Low expression: negative prognostic marker of ACC
SYP	−0.453	3.11 × 10^−6^	Differentiation marker in tumor diagnosis

**Table 2:** Composition of active modules containing one or more of the identified genes of interest listed in Table 1 (in bold) with genes upregulated in red and genes downregulated in blue, representative enrichment and role of differentially expressed genes (DEGs) in cancers.

Module Number and Composition	Representative Enrichment	Role of the DEGs in Cancer
**1** **ASPN**, **COL5A1**, COL25A1, COL3A1, SFRP4	Extracellular matrix organization	CSC maintenance, EMT
**2** ANXA6, **ANXA1**, **ANXA2**, ANXA2R, GREG1, FUCA1, PADi2, **S100A10**, S100A11	Vesicle transport, cell adhesion	Cancer development and progression, poorer prognosis
**4 and 8** **ADCY2**, S1PR3, S1PR5, EDNRA, GNB4, GNG4, **GRK5**, GNAT3, GPR37L1, PTGER3, SSTR3, ECE1, GRPR, HCRTR2, PROK1	Signaling by GPCR	Regulation of CSC, cancer cell survival, metastasis, poor prognosis
**5** NFATC1, **SNAI2**, ATF3, EGR1, FOSL2, GBP1, GBP2, GBP3, IFIT3, IRF1, IRF6, JUN, XAF1	Regulation of cell population proliferation	Maintenance of mesenchymal phenotype, invasive migration, metastasis, poor prognosis, chemotherapy resistance
**16** **TMSB4X**, ACTN4, ISLR, SERPINE1	Platelet degranulation	EMT induction and metastasis
**17 and 165** **PTCH1**, **SHH**, **GLI2**, CTNNA2, **GAS1**, **BOC**, **CDON**, MEF2D, TNNT1, ZIC1, ACTA2, ACTG2, MTSS1	Hedgehog signaling, cell differentiation, cell fate specification, development	Hedgehog pathway activation, CSC maintenance, tumor cell survival
**20** GFRA2, **GRB10**, GRB7	RET signaling	Stem cell self-renewal
**29** **ABCG2**, CYP26B1 ALDH1A1, CYP2S1	Retinoic acid metabolic process	Chemotherapy resistance, CSC, poor prognosis
**51** EHBP1, GAPVD1, **RAB5A**, **RAB31**, EHD3, MYOF, RAB11, FIP2, RAB38	Endosome membrane, endocytosis	Membrane trafficking, endocytosis, cancer cell motility and invasiveness, cancer development and progression, metastasis
**77** DLX6, GREB1, HIST1, H2AC, CBFB, H2AFZ, ITGBL1, PHC1, **RUNX1**, ZFPM1	Negative regulation of differentiation	Poor patient survival, malignant phenotype and metastasis, tumor progression and development, cell proliferation, migration and EMT
**79** CLTCL1, **DNM1**, HIP1, SYT2, **DNM3**	Endocytosis, membrane trafficking	Proliferation, migration and invasion, cancer progression and metastasis, poor prognosis
**85** CEP85 SOX13 **SOX5**	Transcription factors, cell-cycle progression	CSC maintenance, EMT, invasion, metastasis
**89** **CALCR**, FOXE1, GPR176, PTHLH, TSHR, VIPR1	G protein-coupled receptor activity	Stem cell maintenance
**98** CDH10, **CDH2**, CDH12, CDH24, JUP	Cell–cell adhesion	EMT, cell migration and invasion, poor prognosis
**104** CERS4, **CERS6**, **UGCG**, GBA, SMPD4	Sphingolipid metabolism	Multidrug resistance, proliferation of cancer cells, cell migration and metastasis
**106** **MITF**, PMEL, POU3F2	Melanoma phenotype switching	Enrichment of stem cells, de-differentiated state and invasiveness
**127** GBAS, NDUFA8 NDUFS1 PAAF1 UQCRC1 ZNF713, ANKFY1, NDUFA10 **TSPAN11**	Mitochondrial respiratory chain complex	Malignant behavior of cancer cells, cell cycle progression
**178** SLC22A4 SLC29A4 **SLC47A1**	SLC-mediated transmembrane transport	Cell proliferation, epithelial-to-mesenchymal transition

## Supplementary Materials

The following supporting information can be downloaded at: https://www.mdpi.com/article/10.3390/pharmaceutics14050988/s1, Supplementary Materials Table S1: Differential gene expression analysis with DESeq2 was used to estimate the changes between the experimental conditions. Supplementary Materials Table S2: Differentially expressed genes identified by DESeq2 with an adjusted p-value less than 0.05. Supplementary Materials Table S3. Differentially expressed genes identified by DESeq2 with an adjusted p-value less than 0.05 and a log2 fold change greater than 1 or less than −1. Supplementary Materials Table S4. Active gene modules identified by AMINE. Supplementary Materials Table S5. Active gene modules containing one or more genes of interest from Table 1. Supplementary Materials Table S6. Detailed list of enrichments found for each active module from Supplementary Materials Table S3.

## References

[1] Andervont H.B. (1958). Biological Aspects of Cancer. Julian Huxley. Harcourt, Brace, New York, 1958. 156 pp. $3.75. Science.

[2] Johnson B.E., Mazor T., Hong C., Barnes M., Aihara K., McLean C.Y., Fouse S.D., Yamamoto S., Ueda H., Tatsuno K. (2014). Mutational Analysis Reveals the Origin and Therapy-Driven Evolution of Recurrent Glioma. Science.

[3] Prieto-Vila M., Takahashi R.-U., Usuba W., Kohama I., Ochiya T. (2017). Drug Resistance Driven by Cancer Stem Cells and Their Niche. Int. J. Mol. Sci..

[4] Mikubo M., Inoue Y., Liu G., Tsao M.-S. (2021). Mechanism of Drug Tolerant Persister Cancer Cells: The Landscape and Clinical Implication for Therapy. J. Thorac. Oncol..

[5] Balzerano A., Paccosi E., Proietti-De-Santis L. (2021). Evolutionary Mechanisms of Cancer Suggest Rational Therapeutic Approaches. Cytogenet. Genome Res..

[6] Bukowski K., Kciuk M., Kontek R. (2020). Mechanisms of Multidrug Resistance in Cancer Chemotherapy. Int. J. Mol. Sci..

[7] Ozben T. (2006). Mechanisms and strategies to overcome multiple drug resistance in cancer. FEBS Lett..

[8] Wang J.-Q., Yang Y., Cai C.-Y., Teng Q.-X., Cui Q., Lin J., Assaraf Y.G., Chen Z.-S. (2021). Multidrug resistance proteins (MRPs): Structure, function and the overcoming of cancer multidrug resistance. Drug Resist. Updat..

[9] Cree I.A., Charlton P. (2017). Molecular chess? Hallmarks of anti-cancer drug resistance. BMC Cancer.

[10] Kathawala R.J., Gupta P., Ashby C.R., Chen Z.-S. (2015). The modulation of ABC transporter-mediated multidrug resistance in cancer: A review of the past decade. Drug Resist. Updat..

[11] Robey R.W., Pluchino K.M., Hall M.D., Fojo A.T., Bates S.E., Gottesman M.M. (2018). Revisiting the role of ABC transporters in multidrug-resistant cancer. Nat. Rev. Cancer.

[12] Scales S.J., de Sauvage F.J. (2009). Mechanisms of Hedgehog pathway activation in cancer and implications for therapy. Trends Pharmacol. Sci..

[13] Cochrane C., Szczepny A., Watkins D.N., Cain J.E. (2015). Hedgehog Signaling in the Maintenance of Cancer Stem Cells. Cancers.

[14] Hasanovic A., Mus-Veteau I. (2018). Targeting the Multidrug Transporter Ptch1 Potentiates Chemotherapy Efficiency. Cells.

[15] Saze Z., Terashima M., Kogure M., Ohsuka F., Suzuki H., Gotoh M. (2012). Activation of the Sonic Hedgehog Pathway and Its Prognostic Impact in Patients with Gastric Cancer. Dig. Surg..

[16] Xu X., Ding H., Rao G., Arora S., Saclarides C.P., Esparaz J., Gattuso P., Solorzano C.C., A Prinz R. (2012). Activation of the Sonic Hedgehog pathway in thyroid neoplasms and its potential role in tumor cell proliferation. Endocr. -Relat. Cancer.

[17] Bidet M., Tomico A., Martin P., Guizouarn H., Mollat P., Mus-Veteau I. (2012). The Hedgehog Receptor Patched Functions in Multidrug Transport and Chemotherapy Resistance. Mol. Cancer Res..

[18] Hasanovic A., Ruggiero C., Jung S., Rapa I., Signetti L., Ben Hadj M., Terzolo M., Beuschlein F., Volante M., Hantel C. (2018). Targeting the multidrug transporter Patched potentiates chemotherapy efficiency on adrenocortical carcinoma in vitro and in vivo. Int. J. Cancer.

[19] Signetti L., Elizarov N., Simsir M., Paquet A., Douguet D., Labbal F., Debayle D., Di Giorgio A., Biou V., Girard C. (2020). Inhibition of Patched Drug Efflux Increases Vemurafenib Effectiveness against Resistant BrafV600E Melanoma. Cancers.

[20] Lalli E., Luconi M. (2018). The next step: Mechanisms driving adrenocortical carcinoma metastasis. Endocr. Relat. Cancer.

[21] Uchihara M., Tanioka M., Kojima Y., Nishikawa T., Sudo K., Shimoi T., Noguchi E., Maeshima A.M., Yonemori K. (2021). Clinical management and outcomes associated with etoposide, doxorubicin, and cisplatin plus mitotane treatment in metastatic adrenocortical carcinoma: A single institute experience. Int. J. Clin. Oncol..

[22] Edamaghi M., Wojtkowiak J.W., Gillies R.J. (2013). pH sensing and regulation in cancer. Front. Physiol..

[23] Fiorini L., Mus-Veteau I. (2016). Method to Screen Multidrug Transport Inhibitors Using Yeast Overexpressing a Human MDR Transporter. Heterologous Expression of Membrane Proteins.

[24] Durand N., Simsir M., Signetti L., Labbal F., Ballotti R., Mus-Veteau I. (2021). Methiothepin Increases Chemotherapy Efficacy against Resistant Melanoma Cells. Molecules.

[25] Hasanovic A., Simsir M., Choveau F.S., Lalli E., Mus-Veteau I. (2020). Astemizole Sensitizes Adrenocortical Carcinoma Cells to Doxorubicin by Inhibiting Patched Drug Efflux Activity. Biomedicines.

[26] Fiorini L., Tribalat M.-A., Sauvard L., Cazareth J., Lalli E., Broutin I., Thomas O.P., Mus-Veteau I. (2015). Natural paniceins from mediterranean sponge inhibit the multidrug resistance activity of Patched and increase chemotherapy efficiency on melanoma cells. Oncotarget.

[27] Funakoshi K., Bagheri M., Zhou M., Suzuki R., Abe H., Akashi H. (2017). Highly sensitive and specific Alu-based quantification of human cells among rodent cells. Sci. Rep..

[28] Mus-Veteau I. Comparison of Gene Expression between Parental Adrenocortical Carcinoma Cells and a Subpopulation Overexpressing Ptch1. https://www.ncbi.nlm.nih.gov/geo/query/acc.cgi?acc=GSE189424.

[29] Shannon P., Markiel A., Ozier O., Baliga N.S., Wang J.T., Ramage D., Amin N., Schwikowski B., Ideker T. (2003). Cytoscape: A software environment for integrated models of Biomolecular Interaction Networks. Genome Res..

[30] Doncheva N.T., Morris J.H., Gorodkin J., Jensen L.J. (2018). Cytoscape StringApp: Network Analysis and Visualization of Proteomics Data. J. Proteome Res..

[31] Pasquier C., Guerlais V., Pallez D., Rapetti-Mauss R., Soriani O. (2021). Identification of active modules in interaction networks using node2vec network embedding. bioRxiv.

[32] Szklarczyk D., Morris J.H., Cook H., Kuhn M., Wyder S., Simonovic M., Santos A., Doncheva N.T., Roth A., Bork P. (2017). The STRING database in 2017: Quality-controlled protein–protein association networks, made broadly accessible. Nucleic Acids Res..

[33] Grover A., Leskovec J. (2016). node2vec. KDD.

[34] Pasquier C. Active Module Deletion with AMINE Algorithm. http://amine.i3s.unice.fr/.

[35] Izzi L., Lévesque M., Morin S., Laniel D., Wilkes B.C., Mille F., Krauss R.S., McMahon A.P., Allen B.L., Charron F. (2011). Boc and Gas1 Each Form Distinct Shh Receptor Complexes with Ptch1 and Are Required for Shh-Mediated Cell Proliferation. Dev. Cell.

[36] Zhou H.-M., Zhang J.-G., Zhang X., Li Q. (2021). Targeting cancer stem cells for reversing therapy resistance: Mechanism, signaling, and prospective agents. Signal Transduct. Target. Ther..

[37] Najafi M., Farhood B., Mortezaee K. (2019). Cancer stem cells (CSCs) in cancer progression and therapy. J. Cell. Physiol..

[38] Im S., Choi H.J., Yoo C., Jung J.-H., Jeon Y.-W., Suh Y.J., Kang C.S. (2013). Hedgehog Related Protein Expression in Breast Cancer: Gli-2 Is Associated with Poor Overall Survival. Korean J. Pathol..

[39] Papadopoulos V., Tsapakidis K., Galdo N.R.-D., Papandreou C.N., Del Galdo F., Anthoney A., Sakellaridis N., Dimas K., Kamposioras K. (2016). The Prognostic Significance of the Hedgehog Signaling Pathway in Colorectal Cancer. Clin. Color. Cancer.

[40] Zhu W., You Z., Li T., Yu C., Tao G., Hu M., Chen X. (2010). Correlation of Hedgehog Signal Activation with Chemoradio-therapy Sensitivity and Survival in Esophageal Squamous Cell Carcinomas. Jpn. J. Clin. Oncol..

[41] Gonnissen A., Isebaert S., Perneel C., McKee C.M., Van Utterbeeck F., Lerut E., Verrill C., Bryant R.J., Joniau S., Muschel R.J. (2018). Patched 1 Expression Correlates with Biochemical Relapse in High-Risk Prostate Cancer Patients. Am. J. Pathol..

[42] Delloye-Bourgeois C., Gibert B., Rama N., Delcros J.-G., Gadot N., Scoazec J.-Y., Krauss R., Bernet A., Mehlen P. (2013). Sonic Hedgehog Promotes Tumor Cell Survival by Inhibiting CDON Pro-Apoptotic Activity. PLOS Biol..

[43] Mille F., Tamayo-Orrego L., Lévesque M., Remke M., Korshunov A., Cardin J., Bouchard N., Izzi L., Kool M., Northcott P.A. (2014). The Shh Receptor Boc Promotes Progression of Early Medulloblastoma to Advanced Tumors. Dev. Cell.

[44] Song J.Y., Holtz A.M., Pinskey J.M., Allen B.L. (2015). Distinct structural requirements for CDON and BOC in the promotion of Hedgehog signaling. Dev. Biol..

[45] Kaszak I., Witkowska-Piłaszewicz O., Niewiadomska Z., Dworecka-Kaszak B., Toka F.N., Jurka P. (2020). Role of Cadherins in Cancer—A Review. Int. J. Mol. Sci..

[46] Da C., Wu K., Yue C., Bai P., Wang R., Wang G., Zhao M., Lv Y., Hou P. (2016). N-cadherin promotes thyroid tumorigenesis through modulating major signaling pathways. Oncotarget.

[47] Luo S., Lin R., Liao X., Li D., Qin Y. (2021). Identification and verification of the molecular mechanisms and prognostic values of the cadherin gene family in gastric cancer. Sci. Rep..

[48] Chen T.-J., Dehghanian S.Z., Chan T.-C., He H.-L., Li W.-S., Abdollahi S., Chen N.-Y., Li C.-F., Shiue Y.-L. (2021). High G protein subunit beta 4 protein level is correlated to poor prognosis of urothelial carcinoma. Med Mol. Morphol..

[49] Zhou W., Gross K.M., Kuperwasser C. (2019). Molecular regulation of Snai2 in development and disease. J. Cell Sci..

[50] You S., Knudsen B.S., Erho N., Alshalalfa M., Takhar M., Al-Deen Ashab H., Davicioni E., Karnes R.J., Klein E.A., Den R.B. (2016). Integrated Classification of Prostate Cancer Reveals a Novel Luminal Subtype wth Poor Outcome. Cancer Res..

[51] Haslehurst A.M., Koti M., Dharsee M., Nuin P., Evans K., Geraci J., Childs T., Chen J., Li J., Weberpals J. (2012). EMT transcription factors snail and slug directly contribute to cisplatin resistance in ovarian cancer. BMC Cancer.

[52] Li L., Duan Z., Yu J., Dang H.-X. (2016). NFATc1 regulates cell proliferation, migration, and invasion of ovarian cancer SKOV3 cells in vitro and in vivo. Oncol. Rep..

[53] Britzen-Laurent N., Lipnik K., Ocker M., Naschberger E., Schellerer V.S., Croner R.S., Vieth M., Waldner M., Steinberg P., Hohenadl C. (2012). GBP-1 acts as a tumor suppressor in colorectal cancer cells. Carcinogenesis.

[54] Wang J., Min H., Hu B., Xue X., Liu Y. (2019). Guanylate-binding protein-2 inhibits colorectal cancer cell growth and increases the sensitivity to paclitaxel of paclitaxel-resistant colorectal cancer cells by interfering Wnt signaling. J. Cell. Biochem..

[55] Xie G., Dong P., Chen H., Xu L., Liu Y., Ma Y., Zheng Y., Yang J., Zhou Y., Chen L. (2021). Decreased expression of ATF3, orchestrated by β-catenin/TCF3, miR-17-5p and HOXA11-AS, promoted gastric cancer progression via increased β-catenin and CEMIP. Exp. Mol. Med..

[56] Huang C., Chen C., Zheng F., Ni X., Lin J., Wu W., Lai X. (2021). ATF3 inhibits the growth and stem cells-like features of SW620 colorectal cancer cells in vitro. J. Mens. Health.

[57] Shi Q., Yan X., Wang J., Zhang X. (2021). Collagen Family Genes Associated with Risk of Recurrence after Radiation Therapy for Vestibular Schwannoma and Pan-Cancer Analysis. Dis. Markers.

[58] Zhang L., Wang L., Yang H., Li C., Fang C. (2021). Identification of potential genes related to breast cancer brain metastasis in breast cancer patients. Biosci. Rep..

[59] Wang L., Wu H., Wang L., Zhang H., Lu J., Liang Z., Liu T. (2017). Asporin promotes pancreatic cancer cell invasion and migration by regulating the epithelial-to-mesenchymal transition (EMT) through both autocrine and paracrine mechanisms. Cancer Lett..

[60] Itoh G., Takagane K., Fukushi Y., Kuriyama S., Umakoshi M., Goto A., Yanagihara K., Yashiro M., Tanaka M. (2021). Cancer-associated fibroblasts educate normal fibroblasts to facilitate cancer cell spreading and T-cell suppression. Mol. Oncol..

[61] Sasaki Y., Takagane K., Konno T., Itoh G., Kuriyama S., Yanagihara K., Yashiro M., Yamada S., Murakami S., Tanaka M. (2021). Expression of asporin reprograms cancer cells to acquire resistance to oxidative stress. Cancer Sci..

[62] Chi L.-H., Chang W.-M., Chang Y.-C., Chan Y.-C., Tai C.-C., Leung K.-W., Chen C.-L., Wu A.T., Lai T.-C., Li Y.-C. (2017). Global Proteomics-based Identification and Validation of Thymosin Beta-4 X-Linked as a Prognostic Marker for Head and Neck Squamous Cell Carcinoma. Sci. Rep..

[63] Duerr E.-M., Mizukami Y., Ng A., Xavier R.J., Kikuchi H., Deshpande V., Warshaw A.L., Glickman J., Kulke M.H., Chung D.C. (2008). Defining molecular classifications and targets in gastroenteropancreatic neuroendocrine tumors through DNA microarray analysis. Endocr. Relat. Cancer.

[64] Hirata N., Yamada S., Shoda T., Kurihara M., Sekino Y., Kanda Y. (2014). Sphingosine-1-phosphate promotes expansion of cancer stem cells via S1PR3 by a ligand-independent Notch activation. Nat. Commun..

[65] Yang C., Yamashita M., Suda T. (2020). A Novel Function of Sphingolipid Signaling via S1PR3 in Hematopoietic and Leukemic Stem Cells. Blood Cancer Discov..

[66] Watters R.J., Wang H.-G., Sung S.-S., Loughran T.P., Liu X. (2011). Targeting sphingosine-1-phosphate receptors in cancer. Anti-Cancer Agents Med. Chem..

[67] Chen X., Fu Y., Xu H., Teng P., Xie Q., Zhang Y., Yan C., Xu Y., Li C., Zhou J. (2017). SOX5 predicts poor prognosis in lung adenocarcinoma and promotes tumor metastasis through epithelial-mesenchymal transition. Oncotarget.

[68] Tanaka H., Kanda M., Miwa T., Umeda S., Sawaki K., Tanaka C., Kobayashi D., Hayashi M., Yamada S., Nakayama G. (2021). G-protein subunit gamma-4 expression has potential for detection, prediction and therapeutic targeting in liver metastasis of gastric cancer. Br. J. Cancer.

[69] Laurberg J.R., Jensen J.B., Schepeler T., Borre M., Ørntoft T.F., Dyrskjøt L. (2014). High expression of GEM and EDNRA is associated with metastasis and poor outcome in patients with advanced bladder cancer. BMC Cancer.

[70] Kashiwagi E., Shiota M., Yokomizo A., Itsumi M., Inokuchi J., Uchiumi T., Naito S. (2013). Prostaglandin receptor EP3 mediates growth inhibitory effect of aspirin through androgen receptor and contributes to castration resistance in prostate cancer cells. Endocr. Relat. Cancer.

[71] Fa J. (2021). Dynamin 3 overexpression suppresses the proliferation, migration and invasion of cervical cancer cells. Oncol. Lett..

[72] Hu M., Gu J., Su W., Zhang Z., Zhu B., Wang Q., Xing C. (2021). DNM1: A Prognostic Biomarker Associated with Immune Infiltration in Colon Cancer—A Study Based on TCGA Database. BioMed Res. Int..

[73] Rao D., Hyun T.S., Kumar P.D., Mizukami I.F., Rubin M.A., Lucas P., Sanda M.G., Ross T.S. (2002). Huntingtin-interacting protein 1 is overexpressed in prostate and colon cancer and is critical for cellular survival. J. Clin. Investig..

[74] Sheridan M., Ogretmen B. (2021). The Role of Ceramide Metabolism and Signaling in the Regulation of Mitophagy and Cancer Therapy. Cancers.

[75] Schömel N., Gruber L., Alexopoulos S.J., Trautmann S., Olzomer E.M., Byrne F.L., Hoehn K.L., Gurke R., Thomas D., Ferreirós N. (2020). UGCG overexpression leads to increased glycolysis and increased oxidative phosphorylation of breast cancer cells. Sci. Rep..

[76] Suzuki M., Cao K., Kato S., Mizutani N., Tanaka K., Arima C., Tai M.C., Nakatani N., Yanagisawa K., Takeuchi T. (2020). CERS6 required for cell migration and metastasis in lung cancer. J. Cell. Mol. Med..

[77] Singh R.R., Kunkalla K., Qu C., Schlette E., Neelapu S.S., Samaniego F., Vega F. (2011). ABCG2 is a direct transcriptional target of hedgehog signaling and involved in stroma-induced drug tolerance in diffuse large B-cell lymphoma. Oncogene.

[78] Zhang Q., Zhu M., Cheng W., Xing R., Li W., Zhao M., Xu L., Li E., Luo G., Lu Y. (2014). Downregulation of 425G>A variant of calcium-binding protein S100A14 associated with poor differentiation and prognosis in gastric cancer. J. Cancer Res. Clin. Oncol..

[79] Bharadwaj A., Bydoun M., Holloway R., Waisman D. (2013). Annexin A2 Heterotetramer: Structure and Function. Int. J. Mol. Sci..

[80] Hitchcock J.K., Katz A.A., Schäfer G. (2014). Dynamic reciprocity: The role of annexin A2 in tissue integrity. J. Cell Commun. Signal..

[81] Taylor J.R., Fernandez D.J., Thornton S.M., Skeate J., Lühen K.P., Da Silva D.M., Langen R., Kast W.M. (2018). Heterotetrameric annexin A2/S100A10 (A2t) is essential for oncogenic human papillomavirus trafficking and capsid disassembly, and protects virions from lysosomal degradation. Sci. Rep..

[82] Zhang J., Guo B., Zhang Y., Cao J., Chen T. (2010). Silencing of the annexin II gene down-regulates the levels of S100A10, c-Myc, and plasmin and inhibits breast cancer cell proliferation and invasion. Saudi Med. J..

[83] Shen D., Nooraie F., Elshimali Y., Lonsberry V., He J., Bose S., Chia D., Seligson D., Chang H.R., Goodglick L. (2006). Decreased expression of annexin A1 is correlated with breast cancer development and progression as determined by a tissue microarray analysis. Hum. Pathol..

[84] Chua C.E.L., Tang B.L. (2014). The role of the smallGTPase Rab31 in cancer. J. Cell. Mol. Med..

[85] Zhao Z., Liu X.-F., Wu H.-C., Zou S.-B., Wang J.-Y., Ni P.-H., Chen X.-H., Fan Q.-S. (2010). Rab5a overexpression promoting ovarian cancer cell proliferation may be associated with APPL1-related epidermal growth factor signaling pathway. Cancer Sci..

[86] Yang P.-S., Yin P.-H., Tseng L.-M., Yang C.-H., Hsu C.-Y., Lee M.-Y., Horng C.-F., Chi C.-W. (2011). Rab5A is associated with axillary lymph node metastasis in breast cancer patients. Cancer Sci..

[87] Yang T., Zhiheng H., Zhanhuai W., Qian X., Yue L., Xiaoxu G., Jingsun W., Shu Z., Kefeng D. (2020). Increased RAB31 Expression in Cancer-Associated Fibroblasts Promotes Colon Cancer Progression Through HGF-MET Signaling. Front. Oncol..

[88] Hong D., Fritz A.J., Finstad K.H., Fitzgerald M.P., Weinheimer A., Viens A.L., Ramsey J., Stein J.L., Lian J.B., Stein G.S. (2018). Suppression of Breast Cancer Stem Cells and Tumor Growth by the RUNX1 Transcription Factor. Mol. Cancer Res..

[89] Zhao Z., Liang S., Sun F. (2020). LncRNA DLX6-AS1 Promotes Malignant Phenotype and Lymph Node Metastasis in Prostate Cancer by Inducing LARGE Methylation. Front. Oncol..

[90] Wu Q., Ma J., Meng W., Hui P. (2019). DLX6-AS1 promotes cell proliferation, migration and EMT of gastric cancer through FUS-regulated MAP4K1. Cancer Biol. Ther..

[91] Zhao X., Wang J., Zhu R., Zhang J., Zhang Y. (2021). DLX6-AS1 activated by H3K4me1 enhanced secondary cisplatin resistance of lung squamous cell carcinoma through modulating miR-181a-5p/miR-382-5p/CELF1 axis. Sci. Rep..

[92] Hodgkinson K., Forrest L.A., Vuong N., Garson K., Djordjevic B., Vanderhyden B.C. (2018). GREB1 is an estrogen receptor-regulated tumour promoter that is frequently expressed in ovarian cancer. Oncogene.

[93] Hu J., Tian J., Zhu S., Sun L., Yu J., Tian H., Dong Q., Luo Q., Jiang N., Niu Y. (2017). Sox5 contributes to prostate cancer metastasis and is a master regulator of TGF-β-induced epithelial mesenchymal transition through controlling Twist1 expression. Br. J. Cancer.

[94] Chen Y., Yang L., Qin Y., Liu S., Qiao Y., Wan X., Zeng H., Tang X., Liu M., Hou Y. (2020). Effects of differential distributed-JUP on the malignancy of gastric cancer. J. Adv. Res..

[95] Sun C., Ban Y., Wang K., Sun Y., Zhao Z. (2019). SOX5 promotes breast cancer proliferation and invasion by transactivation of EZH2. Oncol. Lett..

[96] Du F., Li X., Feng W., Qiao C., Chen J., Jiang M., Qiu Z., Qian M., Tian D., Nie Y. (2020). SOX13 promotes colorectal cancer metastasis by transactivating SNAI2 and c-MET. Oncogene.

[97] Jiao H., Fang F., Fang T., You Y., Feng M., Wang X., Yin Z., Zhao W. (2021). SOX13 regulates cancer stem-like properties and tumorigenicity in hepatocellular carcinoma cells. Am. J. Cancer Res..

[98] Yamaguchi M., Watanabe Y., Ohtani T., Uezumi A., Mikami N., Nakamura M., Sato T., Ikawa M., Hoshino M., Tsuchida K. (2015). Calcitonin Receptor Signaling Inhibits Muscle Stem Cells from Escaping the Quiescent State and the Niche. Cell Rep..

[99] Li H., Liu P., Xu S., Li Y., Dekker J.D., Li B., Fan Y., Zhang Z., Hong Y., Yang G. (2017). FOXP1 controls mesenchymal stem cell commitment and senescence during skeletal aging. J. Clin. Investig..

[100] Simmons J., Pierce C.J., Al-Ejeh F., Boyle G.M. (2017). MITF and BRN2 contribute to metastatic growth after dissemination of melanoma. Sci. Rep..

[101] Carreira S., Goodall J., Denat L., Rodriguez M., Nuciforo P., Hoek K.S., Testori A., LaRue L., Goding C.R. (2006). Mitf regulation of Dia1 controls melanoma proliferation and invasiveness. Genes Dev..

[102] Travnickova J., Wojciechowska S., Khamseh A., Gautier P., Brown D.V., Lefevre T., Brombin A., Ewing A., Capper A., Spitzer M. (2019). Zebrafish MITF-Low Melanoma Subtype Models Reveal Transcriptional Subclusters and MITF-Independent Residual Disease. Cancer Res..

[103] Hoek K.S., Goding C.R. (2010). Cancer stem cells versus phenotype-switching in melanoma. Pigment. Cell Melanoma Res..

[104] Johannes L. (2021). The Cellular and Chemical Biology of Endocytic Trafficking and Intracellular Delivery—The GL–Lect Hypothesis. Molecules.

[105] Christenson L. (2000). Steroidogenic acute regulatory protein (StAR) and the intramitochondrial translocation of cholester-ol. Biochim. Biophys. Acta Mol. Cell Biol. Lipids.

[106] Wiedenmann B., Franke W.W., Kuhn C., Moll R., E Gould V. (1986). Synaptophysin: A marker protein for neuroendocrine cells and neoplasms. Proc. Natl. Acad. Sci. USA.

[107] Faillot S., Assie G. (2016). ENDOCRINE TUMOURS: The genomics of adrenocortical tumors. Eur. J. Endocrinol..

[108] Brondani V., Lacombe A., Mariani B., Montenegro L., Soares I., Bezerra-Neto J., Tanno F., Srougi V., Chambo J., Mendonca B. (2021). Low Protein Expression of both *ATRX* and *ZNRF3* as Novel Negative Prognostic Markers of Adult Adrenocortical Carcinoma. Int. J. Mol. Sci..

[109] Bai X., Fisher D.E., Flaherty K.T. (2019). Cell-state dynamics and therapeutic resistance in melanoma from the perspective of MITF and IFNγ pathways. Nat. Rev. Clin. Oncol..

